# A Bio-Guided Screening for Antioxidant, Anti-Inflammatory and Hypolipidemic Potential Supported by Non-Targeted Metabolomic Analysis of *Crepis* spp.

**DOI:** 10.3390/molecules27196173

**Published:** 2022-09-20

**Authors:** Christina Barda, Konstantina Anastasiou, Ariadni Tzara, Maria-Eleni Grafakou, Eleftherios Kalpoutzakis, Joerg Heilmann, Michael Rallis, Angeliki P. Kourounakis, Helen Skaltsa

**Affiliations:** 1 Department of Pharmacognosy & Chemistry of Natural Products, Faculty of Pharmacy, School of Health Sciences, National & Kapodistrian University of Athens, 15771 Athens, Greece; 2 Department of Pharmaceutical Biology, Faculty of Pharmacy and Chemistry, University of Regensburg, D-93053 Regensburg, Germany; 3 Department of Medicinal Chemistry, Faculty of Pharmacy, School of Health Sciences, National & Kapodistrian University of Athens, 15771 Athens, Greece; 4 Unity of Dermatopharmacology, Department of Pharmaceutical Technology, Faculty of Pharmacy, School of Health Sciences, National & Kapodistrian University of Athens, 15771 Athens, Greece

**Keywords:** *Crepis*, Asteraceae, LC-MS, NMR, cichoric acid, phenolic acid, biological activity, mouse paw edema, antihyperlipidemic

## Abstract

This study was designed to evaluate the chemical fingerprints and the antioxidant, anti-inflammatory and hypolipidemic activity of selected *Crepis* species collected in Greece, namely, *C. commutata, C. dioscoridis, C. foetida, C. heldreichiana, C. incana, C. rubra,* and *Phitosia crocifolia* (formerly known as *Crepis crocifolia*). For the phytochemical analyses, sample measurements were carried out by using nuclear magnetic resonance (NMR) spectroscopy and liquid chromatography coupled with mass spectrometry (LC-MS). Τhe extracts were evaluated both in vitro (radical scavenging activity: DPPH assay and total phenolic content: Folin–Ciocalteu) and in vivo (paw edema reduction and hypolipidemic activity: experimental mouse protocols). Among the tested extracts, *C. incana* presented the highest gallic acid equivalents (GAE) (0.0834 mg/mL) and the highest antioxidant activity (IC_50_ = 0.07 mg/mL) in vitro, as well as the highest anti-inflammatory activity with 32% edema reduction in vivo. Moreover, in the hypolipidemic protocol, the same extract increased plasma total antioxidant capacity (TAC) by 48.7%, and decreased cholesterol (41.3%) as well as triglycerides (37.2%). According to fractionation of the extract and the phytochemical results, this biological effect may be associated with the rich phenolic composition; caffeoyl tartaric acid derivatives (cichoric and caftaric acid) are regarded as the most prominent bioactive specialized metabolites. The present study contributes to the knowledge regarding the phytochemical and pharmacological profile of *Crepis* spp.

## 1. Introduction

The genus *Crepis* L. (Asteraceae) comprises more than 200 currently recognized species, of which less than 10% have been investigated, either from a phytochemical or bioactivity point of view [1,2]. The genus has been reported to be rich in phenolics and flavonoids, with predominant compounds being caffeoyl and luteolin derivatives, respectively [1]. Another widespread group of specialized natural products are sesquiterpene lactones and especially guaianolides, with other terpenoids also being identified, such as triterpenes and sterols [3]. The consensus view of specialized herbal metabolites as promising medicinal agents for preventing or managing oxidative stress, inflammation, hyperlipidemia and other related disorders, has been shown by maintaining good health and retarding aging processes [4]. Even though phytoconstituents of the *Crepis* genus have been documented, its pharmacological potential has not been fully explored yet. Extracts obtained from the different parts of the *Crepis* spp. and their isolated constituents have undergone only limited evaluation as antitumor, anti-inflammatory, antiviral, antimicrobial, antiulcer, antioxidant and nutritional agents [5,6]. Moreover, within the *Crepis* genus, few edible representatives appear, such as *C. bulbosa*, *C. capillaris*, *C. commutata*, *C. foetida*, *C. setosa* and *C. vesicaria* [5,6]. In Greece and Italy, many *Crepis* spp. are widely consumed together with other edible green herbs as an integral part of the traditional Mediterranean diet [6]. In this context, seven species were collected from different areas around Greece, specifically *C. commutata*, *C. dioscoridis*, *C. foetida*, *C. heldreichiana*, *C. incana*, *C. rubra*, and *Phitosiacrocifolia* (formerly known as *Crepiscrocifolia*). These samples were extracted and further investigated for their chemical profile in correlation with their biological effects. It is worth mentioning that among the selected plants, *C. heldreichiana*, *C. incana*, and *Phitosiacrocifolia* account for narrowly endemic types with limited distribution.

A non-targeted metabolomic strategy was selected to analyze and determine the differences in the phytochemical fingerprints of the abovementioned extracts. In detail, sample measurements were carried out by using both nuclear magnetic resonance (NMR) spectroscopy and liquid chromatography coupled with mass spectrometry (LC-MS). Among the under-investigation species, there are no reports in the literature except for *C. commutata*, *C. incana* and *C. dioscoridis*, which have been previously described by our group with emphasis on the non-polar and/or less polar sesquiterpene lactones [5,7,8]. In addition, we, herein, examined the polar extracts, which are more likely to contain hydrophilic compounds more relevant to their local use as edible green herbs and potentially potent antioxidant agents. Thus, the present study further includes a bio-guided approach using antioxidant, anti-inflammatory and hypolipidemic assays to unveil the most promising plant extract(s) through both in vitro and in vivo experimental procedures. Accordingly, the presented data encompass a widely applicable strategy for screening plant extracts, as well as information on the chemical and biological effects of the genus *Crepis*, regarding previously uninvestigated plant species. Moreover, to the best of our knowledge, no other comprehensive pharmacological evaluation has been reported so far.

## 2. Results and Discussion

### 2.1. Phytochemical Characterization of Selected Extracts

NMR and LC-MS analyses can be powerful tools to assess the chemical composition of specialized natural products that occur in complex mixtures. In respect to NMR, such protocols involve a “metabolomic” approach supported by a plethora of previously published papers, displaying the usefulness of both 1D and 2D NMR experiments for compound identification in complex matrix-like plant extracts, and also for comparative and qualitative analysis, as well as several other purposes and applications [9]. The relevant advantage of this approach is attributed to the unique NMR property of having the same response factor for different classes of metabolites. This can be used complementary to LC-MS analyses, as the sensitivity and selectivity with the low limit of detection (LODs)of LC-MS measurements (LODs 10^−13^ mol) are unachievable through NMR, thus, their combination is particularly advantageous. In this study, this approach was applied to different *Crepis* extracts to detect various classes of constituents by NMR and LC-MS/MS [10]. For the phytochemical analysis, the plant materials were initially extracted with MeOH:H_2_O and further subjected to liquid/liquid extraction with n-butanol: H_2_O, in order to achieve the elimination of sugars and obtain extracts rich in small molecules. The results provided by the phytochemical analyses were further associated with the data obtained from their biological evaluation.

#### 2.1.1. NMR Characterization of Selected n-Butanol Extracts

**Figure 1:**
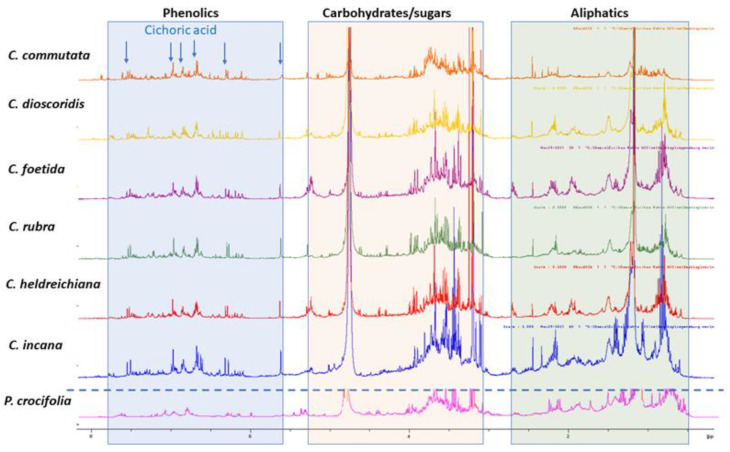
^1^H NMR comparison of plant n-butanol extracts: *Crepiscommutata*(orange), *C. dioscoridis* (yellow), *C. foetida* (purple), *C. rubra* (green), *C. heldreichiana* (red), *C. incana* (blue), *Phitosiacrocifolia* (pink) in CD_3_OD; diagnostic signals for cichoric acid are indicated with blue arrows.

An overlay of the^1^H-NMR spectra acquired for the selected plant n-butanol extracts is depicted in Figure 1, where the regional differentiation has been highlighted in different colors, aiming to distinguish the corresponding signals for phenolic derivatives (blue background), hydrocarbons, sugars and terpenoids (orange background), as well other signals assigned in the aliphatic region (green background). Moreover, due to many overlapping peaks, 2D NMR was utilized for further characterization of the ingredients. The 2D NMR spectroscopic data of all samples are provided in SI. Moreover, in Figure 1,^1^H NMR diagnostic signals for characteristic compounds (cichoric acid and/or caftaric acid) have also been indicated (arrows).

In detail, visual inspection of the 1D and 2D NMR spectra revealed the presence of different classes of metabolites. Starting with the region 5.5 to 8.5 ppm, phenolic derivatives are observed, and especially cichoric acid is predominant in all samples apart from *P. crocifolia* and *C. commutata*. Other caffeoyl derivatives, such as caffeoyl quinic isomers and flavonoids, are also detected in this region. Cichoric acid (2,3-di-*O*-caffeoyltartaric acid) was identified on the basis of 1D and 2D NMR spectroscopic data. Specifically, ^1^H NMR spectra of the n-butanol extracts exhibit signals belonging to its two caffeic moieties with two trans olefinic protons at 7.47 (2H, d, *J* = 15.0 Hz, H-7′, 7″) and 6.27 (2H, d, *J* = 15.0 Hz, H-8′, 8″), and three aromatic protons of the ABX pattern at 7.05 (2H, s, H-2′, 2″), 6.94 (2H, s, H-6′, 6″) and 6.75 (2H, s, H-5′, 5″). A singlet at 5.54 ppm is attributed to the tartaric acid moiety of the compound [11]. Heteronuclear correlation experiments (HSQC and HMBC) provide additional data for structure identification (see Supplementary Material (SM)). As the extracts are complex mixtures due to the existence of other substances, the observed chemical shifts (δ_H_ values) in their ^1^H NMR spectra, compared to the spectra of the pure compounds, reveal a deviation of ±0.05 ppm.

Among the measured samples, *C. commutata* possesses a lower amount of cichoric acid based on the signal of the tartaric acid moiety. Moreover, cichoric acid was not detected in *P. crocifolia*. It is worth mentioning that the main phenolics of *P. crocifolia* were inconsistent with caffeoyl quinic acid isomers and caffeic acid methyl ester (two doublets with J = 16.0 Hz at δ_H_ 7.53 and 6.22; ABX system with dJ = 2.1 Hz at δ_H_ 7.03, dJ = 8.2 Hz at 6.77 and dd J = 8.2, 2.1 Hz at6.93; singlet at δ_H_3.75) [12]. Of note, in *P. crocifolia*, sesquiterpene lactones were present in equal proportions with the phenolic derivatives, in contrast to the other samples. The NMR data permitted the identification of 2β-hydroxysantamarine-1β-D-glucopyranoside, an eudesmane-type sesquiterpene lactone previously reported by Zidorn et al. [13] from Taraxacum linearisquameum, as well as guaianolides with vinylic methyl at C14 andC15 (singlet at δ_H_ca.2.40 and ca. 2.30, respectively).

In HSQC, signals supporting the presence of flavonoid O-glycosides can be observed, such as protons of the substituted aromatic (B-) ring and several signals ascribable to H-6 and H-8, as well H-3 of the chromanone nucleus. Nevertheless, the density of the aromatic signals of flavonoids in the HSQC experiment is more distinct for the widely distributed *C. commutata*, *C. dioscoridis*, *C. rubra* and *C. foetida* (see Supplementary Materials) in comparison to the narrow endemic species (*C. incana* and *P. crocifolia*). Flavonoid accumulation can be affected by several factors, including the increase in temperature and UV-B radiation, and varies among organs within plants [14].

In higher fields, and especially in the 3–5 ppm overcrowded region, signals for different types of free sugars or sugar moieties of glucosides appear. As depicted in Figure 1, carbohydrate and glycoside substituents can be observed due to the partially overlapped doublets in the range 4.7–5.10 ppm, showing HSQC-DEPT correlations with CH carbons in the range of δ_C_ 90–105, that support the presence of the anomeric position of sugars.

Lastly, in the aliphatic region (0.6–2.5 ppm), the NMR analyses of all extracts confirmed the presence of fatty acid esters. The multiplets at δ_H_ca. 5.34 were attributed to the olefinic protons (-CH=CH-) of the unsaturated fatty esters and the triplets at δ_H_ ca. 0.88 and/or 0.95 (J = 6.9) were attributed to the terminal CH_3_ group of the alkyl chain; these signals were observed over a wide range of chemical shifts due to the degree of unsaturation. The nearby -CH_2_ of the esters (-COOR) were distributed at δ_H_ ca. 2.33 (t, J = 6.8), and the intermediate -CH_2_- of the double bonds were resonated at δ_H_ ca. 2.76 (m). The rest -CH_2_ of the fatty ester chains were assigned to the intense signal at δ_H_ ca. 1.24.

#### 2.1.2. LC-MS Characterization of Selected n-Butanol Extracts

To gain further insight into the chemical composition, the n-butanol extracts of the selected plants were submitted to LC-MS/MS analysis. Based on these results, the main constituents of *Crepis* spp. were confirmed to be caffeoyl tartaric acid derivatives. Moreover, the LC-MS analysis revealed the presence of more than 52 compounds, including sugars, sesquiterpene lactones and phenolics (flavonoids, phenolic acids and other phenolic derivatives). In Table 1, compounds are listed according to their retention time. The chemical characterization was in agreement with previously published data on the Cichorieae tribe and the Asteraceae family. For the identification of the compounds, the first step was performed by building an in-house database with approximately 200 molecules that have been described previously in the *Crepis* genus. In addition, all information was interpreted and correlated with mass spectra available in the literature and online databases. The molecular formulas were confirmed based on high-precision quasi-molecular ions such as [M-H]^−^, [M+CHCOO]^−^, [M+HCOO]^−^, [M+H]^+^ or [M+Na]^+^ with a mass error of 5.0 ppm. The monitoring of phenolics and other compounds in the negative mode is reported to be more sensitive for the analysis [15]. Furthermore, the MS/MS spectra allowed an additional level of identification through the fragmentation patterns.

**Figure 2:**
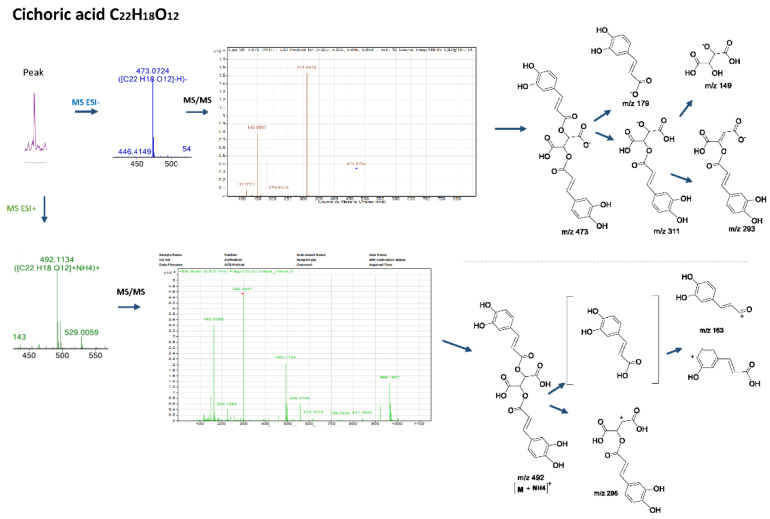
Mass spectra, fragmentation patterns and suggested molecular structures of diagnostic ions in positive and negative ion modes for cichoric acid.

More specifically, the LC-MS results revealed the presence of the two main caffeoyl tartaric acid derivatives. Cichoric acid with a mass of 473.0719 [M-H]^−^, followed by a peak at *m/z* 311 [M-163-H]^−^ due to the loss of a caffeoyl- unit, a peak at *m/z* 293 [M-179-H]^−^due to the loss of a caffeic acid, followed by the fragments *m/z* 179 and *m/z* 149 of tartaric acid, which resulted from the loss of the two caffeoyl-units, is consistent with the mass spectrum for the title compound and the molecular formula C_22_H_18_O_12_ [15,16]. In Figure 2, the fragmentation patterns and the suggested molecular structures of diagnostic ions are presented for the positive and negative ion modes. Similarly, caftaric acid (C_13_H_11_O_9_) with a precursor ion of *m/z* 311.0405, fragmented to produce ions at *m/z* 149, *m/z* 179 and *m/z* 135 [179-CO_2_]^−^which resulted from decarboxylation of the caffeic acid residue confirmed the presence of this compound [17].

Mono-acyl chlorogenic acid isomers matched with compounds with a molecular ion [M-H]^−^ at *m/z* 353 and chemical formulas C_16_H_18_O_9_, and, with respect to the literature, were attributed to 5- or 3- caffeoylquinic acids isomers (*m/z* 353.0870), producing fragment ions at *m/z* 191 [M-163-H]^−^due to the loss of a caffeoyl moiety [18].

Flavonoid derivatives were also present in the extracts, with luteolin isomers, as aglycones or as glycosides, such as luteolin isomers *m/z* 286.0478 (C_15_H_10_O_6_, *m/z* 285.0405 [M-H]^−^), luteolin glycoside isomers *m/z* 448.1009 (C_21_H_20_O_11_, *m/z* 447.0935 [M-H]^−^) and luteolin diglycoside isomers *m/z* 610.1528 (C_27_H_30_O_16,_ *m/z* 609.1454 [M-H]^−^) being the most prominent [19].

Sesquiterpene lactones play a significant role in plants and their bioactivity and structural diversity are well studied [20]. Nevertheless, their nature limits the applicability of UV detection, as they lack chromophoric groups. Moreover, the ionization of sesquiterpene lactones has been recorded to be more effective in the positive mode in [M+H]^+^, [M+Na]^+^ or results in ions formed from solvent adducts and/or the loss of water or acid residues, facts that prevent their flawless detection [21]. On top of this, databases and the literature are lacking data for their fragmentation patterns, since many of them have been recorded only once. Considering the above, for the annotation of sesquiterpene lactones, ions of [M+H]^+^ and [M+Na]^+^ were detected and led to the chemical formulas that possessed a 15-carbon backbone or C-21 (C-15 and hexose) or C-n (C21 and various phenolic substituents) based on literature data and with the aid of the NMR spectra.

It is noteworthy that the accumulation of specialized metabolites during plant development is highly affected by many interdependent factors, such as light, photoperiod, temperature, soil water, salinity or fertility, etc. [14]. Especially, phytochemical studies for wild species feature a chemical fingerprint influenced by environmental and physiological parameters based on the time of collection. The biochemical adaptability in plants is relatively hard to discover and is still not fully understood.

#### 2.1.3. Total Phenolic Content

For the quantification of phenolic compounds in the selected plant extracts and fractions, the Folin–Ciocalteu (F-C) method was selected, as it is a rapid method, widely applied for the determination of total polyphenol content (TPC) and it is quoted by several pharmacopoeias [22]. Polyphenols react with the F-C reagent, leading to blue-colored complexes that absorb at a specific wavelength. For this quantification, a standard solution of gallic acid was used for the reference curve, for measuring the phenolic content for each sample. TPC estimated by F-C assay ranged from 0.0359 to 0.0834 mg/mL for the hydroalcoholic and aqueous extracts, while the fractions showed similar levels from 0.1210 to 0.0937 mg/mL (Table 2).

### 2.2. In Vitro and In Vivo Activities

One of the greatest challenges concerning the chemical analysis of multiple extracts is that metabolites must be studied individually and in detail to obtain qualitative characteristics, which include time-consuming and expensive techniques. In this aspect, in vitro bio-guided tests can provide relevant information to allow the researchers to decide whether to select and intensify the fractionation and purification of established bioactive samples. This experimental approach permitted us the selection of *C. incana*, *C. heldreichiana* and *C. dioscoridis* based on their in vitro effects. Initially, the antioxidant activity and the determination of the phenolic content (as discussed above) were evaluated in nine complex samples, in which the most prominent was selected and relevant fractionation was conducted to diminish its complexity. The yielded fractions were similarly evaluated to unveil the active sub-fraction that demonstrates the biological effects of interest. The next step involved the in vivo assessment of the extracts and obtained fractions, on induced inflammation and hyperlipidemia in respective experimental mouse models.

The key role of free radical reactions in pathological abnormalities and their involvement in many acute and chronic inflammatory disorders, including atherosclerosis and diabetes, is well documented [23,24,25]. Thus, by addressing oxidative stress, inflammationand dyslipidemia with the potentially multifunctional nature of specialized natural products, we aimed to identify agents, such as extracts or compounds, that could possibly serve against various such related disorders.

#### 2.2.1. Evaluation of Antioxidant Activity In Vitro, DPPH Assay

Using the DPPH assay, the antioxidant ability of different extracts and fractions of plants belonging to *Crepis* genus, expressed as IC_50_ values, compared to the reference compounds resveratrol (RSV), ascorbic acid (ASC) and di-t-butylhydroxytoluene (BHT), are shown in Table 3. MeOH:H_2_O 5:1 extracts showed IC_50_ values of 0.07 to 0.26 mg/mL, while H_2_O (100%) extracts possessed higher IC_50_ values of 0.30 to >1 mg/mL. As expected, the aqueous extracts rich in sugars showed the lowest activity in comparison tothe hydroalcoholic extracts. Among them, the hydroalcoholic extract of *C. incana* showed the most potent antioxidant activity with IC_50_ 0.07 mg/mL (Table 2) and, therefore, was selected for further fractionation; the resulted fractions were evaluated in the same assay. Fractions RINH (yielded with EtoAc:MeOH:H_2_O 2:8:0.8), RINI (yielded with MeOH 100%) and RINJ (yielded with MeOH:H_2_O 1:1) were proven active. RINI was the most potent antioxidant fraction, with the lowestobservedIC_50_ value (0.06 mg/mL), compared to the other fractions and the parent extract (*C. incana* MeOH:H_2_O 5:1). The activity of *C. incana* 5:1 and RINI was comparable or even greater than reference compounds (RSV IC_50_ 0.02 mg/mL; ASC IC_50_ 0.01 mg/mL; BHT IC_50_ 0.38 mg/mL). As seen, this assay demonstrated that the genus Crepis is a great source of antioxidant compounds responsible for radical scavenging ability; in particular, this effect was attributed to their phenolic content.

DPPH IC_50_ values (as an indicator of in vitro antioxidant capacity) were tested for possible correlation with the TPC values for each extract/fraction. This revealed that the extracts MeOH:H_2_O 5:1 have a higher antioxidant potency (lower IC_50_ values) and higher gallic acid equivalents (GAE) or TPC. In detail, the most active was *C. incana* 5:1, bearing the highest GAE (0.0834 mg/mL) and the lowest IC_50_ value (0.07 mg/mL) among the tested plant extracts. The most active fractions, RINB, RINC and RINI, showed the highest TPC values, among which RINI also had the lowest IC_50_ (0.06 mg/mL), i.e., the best antioxidant activity.

#### 2.2.2. Evaluation of Anti-Inflammatory Activity (In Vivo)

**Figure 3:**
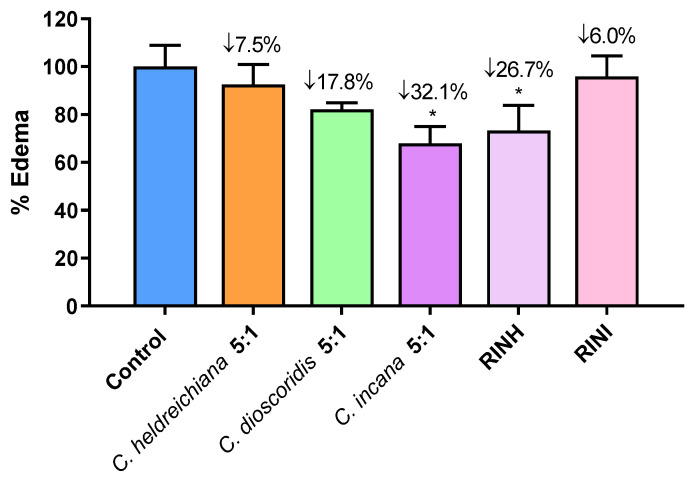
Effect of extracts *C. heldreichiana* 5:1, *C. dioscoridis* 5:1, *C. incana* 5:1 and fractions RINH and RINI on carrageenan-induced mouse paw edema. Each value represents the mean obtained from 6 animals. Significant difference of Control from *C. incana* group: * *p* = 0.05 and from RINH: * *p* = 0.05. [The average weight difference between the injected and un-injected paws for the Control group (100%) is 0.058 g (mean weight of injected/uninjected paw is 0.210 vs. 0.146 g)].

The carrageenan-induced paw edema is a well-known acute model of inflammation that is widely used for screening novel anti-inflammatory agents [25,26,27]. Based on the in vitro results, the most active extracts and fractions were selected and further evaluated for their anti-inflammatory activity at a dose of 3 mg/100 g of body weight i.p. using the carrageenan-induced paw edema assay. Results are shown in Figure 3 and are expressed as % edema reduction. It is worth mentioning that the extract of *C. incana* 5:1 and the fraction RINH caused a statistically significant edema reduction, with *C. incana* 5:1 bearing the highest anti-inflammatory activity (32% edema reduction) in vivo and in the activity range of known anti-inflammatory agents e.g., Indomethacin: 29%, Ibuprofen: 36% at a dose of 1.5 mmol/100 g of body weight, respectively [28,29]. It seems that the high antioxidant activity of these extracts/fractions (combined with an apparently optimum bioavailability) may be contributing to their anti-inflammatory effect [25,28,30].

#### 2.2.3. Evaluation of (In Vivo) Antioxidant and Anti-Hyperlipidemic Activity

**Figure 4:**
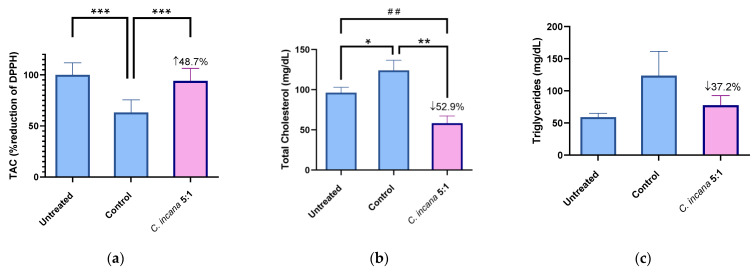
Representative graphs for TAC (**a**), TC (**b**) and TG (**c**) of the most active extract *C. incana* 5:1, compared to Untreated and Control group. Significant difference in (**a**) of Control group from Untreated group: *** *p* = 0.0001, *C. incana* 5:1 from Control group: *** *p* = 0.0001. Statistical difference in (**b**) of Control from Untreated group: * *p* = 0.045. *C. incana* 5:1 from Control: ** *p* = 0.001, from Untreated group: ## *p* = 0.0045.

The extracts/fractions studied in this protocol were administered to mice i.p. (dose: 0.3 mg/kg body weight) after tyloxapol i.p. administration, which caused acute hyperlipidemia. The total antioxidant capacity (TAC), total cholesterol (TC) and triglyceride levels (TG) in plasma were measured and compared to control and untreated groups, 24 h after tyloxapol administration. Results are shown in Table 4, Table 5 and Table 6, while representative graphs of the effect of the most active extract *C. incana* 5:1 are shown in Figure 4.

The most active extract that increased total antioxidant capacity (TAC) by 49%, compared to (the hyperlipidemic) Control, was *C. incana* 5:1 (Figure 4a). In specific, it almost restored TAC levels to normal (6% lower than the Untreated group). At the same time, it was one of the two most active extracts in lowering TC levels (53%), together with *C. dioscoridis* 5:1 (57%) (Figure 4b). Regarding TG levels, *C. incana* 5:1 showed again the highest potency, decreasing TG levels by 37% (Figure 4c). Thus, the antioxidant profile of *C. incana* 5:1 was superior to the other extracts/fractions. Even though *C. dioscoridis* 5:1 was successful at lowering TC levels, it did not show such a similar potency in TAC or TG levels. It is concluded that *C. incana* 5:1 caused a significant decrease in TC and TG levels, as well as the greatest antioxidant (TAC) and anti-inflammatory (edema) activity in vivo, proving the most promising extract for further studies.

### 2.3. Pharmacological Profile of Crepis *spp.*

The genus *Crepis* belongs to the tribe Cichorieae, which encompasses many well-known taxa such as *Taraxacum* spp. and *Cichorium* spp. [1]. According to the literature, a plethora of records illustrate their significant pharmacological interest [31,32]. Nevertheless, the biological potential of the genus *Crepis* is untapped. As described previously, the chemical compositions of the *Crepis* taxa mainly includes phenolics and flavonoids as well as lignans, sterols, volatile constituents, triterpenesand sesquiterpene lactones, of which phenolics andsesquiterpene lactones are generally considered the major active components [6,33].Contemporary pharmacological studies aim at evaluating *Crepis* extracts or their isolated compounds with in vitro assays, while in vivo experiments related to this genus are more or less absent.Limited records for antibacterial, antifungal, antiviral, cytotoxic and antiulcer activitiesand toxicity, as well as (relevant to our study) antioxidant, anti-inflammatoryandantidiabetic potential can be found for the *Crepis* taxa [6]. In detail, a previous study showed the antioxidant activity of the aerial parts from *C. foetida* L. subsp. *rhoeadifolia* using DPPH (IC_50_ = 0.26 mg/mL) and TBARS assays (MDA level = 4.54 nmol/mL) [34]. The methanolic extract of *C. sancta* [35] was evaluated for its antioxidant capacity with the ABTS decolorization assay and alsoshowed promising results, while the infusion fromaerial parts of *C. foetida* L. [35] showed moderate activity (DPPH method). Lastly, the hydro–alcoholic extract of *C. vesicaria* subsp. *taraxacifolia* exhibited antioxidant activity in DPPH (IC_50_ = 26.20 μg/mL), ABTS (IC_50_ = 18.92 μg/mL) and FRAP (0.68 μg/mL of Trolox Equivalent) [36].

Based on our biological evaluation by use of the DPPH methodregarding *C. commutata*, *C. dioscoridis*, *C. foetida*, *C. heldreichiana*, *C. incana*, *C. rubra*, *C. commutata*, and *Phitosia crocifolia*, the aqueous extract possessed lower activity, in accordance with the literature, while the tested methanol–water extracts possessed higher DPPH inhibition in the range of IC_50_= 0.07 to 0.26 mg/mL. According to our phytochemical findings, these antioxidant effects can be associated with the rich phenolic composition, i.e., caffeoyl tartaric acid derivatives (cichoric and caftaric acid), other caffeoyl derivatives and flavonoids. *C. incana* revealed the most prominent activity, while its bio-active subfractions suggested the compounds responsible for the observed effects. Fractions RINI with IC_50_ value 0.06 mg/mL, characterized by cichoric acid, while fractions RINB-RINE with IC_50_ values ranging from 0.12 to 0.35 mg/mL showed high amounts of flavonoid glucosides (luteolin–quercetin derivatives), along with free sugars and minor levels of sesquiterpene lactones.Based on the literature, several phenolics, including caftaric acid, chlorogenic acid, caffeic acid, cichoric acid, 3,5-di-O-caffeoylquinic acid and luteolin, isolated from *Taraxacum mongolicum*, have been reported to exert IC_50_ values of 7.3, 12.7, 7.1, 9.6, 10.1, 5.3 µg/mL, respectively (DPPH method) [37].

To the best of our knowledge, one study showed the anti-inflammatory activity of *C. vesicaria* subsp. *taraxacifolia* (ethanolic extract) in vitro (using macrophage cell cultures) by means of inhibition of NO production in a dose-dependent manner with an IC_50_ of 0.43 mg/mL [36]. In our findings, the in vivo anti-inflammatory activity at a dose of 0.3 mg/kg of body weight i.p. via the reduction in carrageenan-induced paw edema of the methanol:water extract of *C. incana* and the fraction RINH showed statistically significant results, exerting up to 32% edema reduction. For comparison purposes, it is worth noting that in another study, *Taraxacum officinale*, administered orally (100 mg/kg bodyweight) 1 h before edema, elicited inhibition of carrageenan-induced rat paw edema by 25% [31]. The different administration routes (gastrointestinal or intraperitoneal) may give rise to the argument of bioavailability of cichoric acid and similar phenolic derivatives [16], which may hamper their clinical application. Contradictory reports show low permeability per os and the possibility of transfer across the blood-brain barrier [16,17,38]. Additional in vivo research demonstrated that cichoric acid was partly metabolized through Phase I to caffeic acid and caftaric acid by cytochrome P450s in rat liver [17].

Cardiovascular diseases and diabetes are related to metabolic disorders that include hyperglycemia, hyperlipidemia, hyperuricemia, hypertension, atherosclerosis, etc. [38]. Many studies have been performed on species from the tribe Cichorieae, whichare widely consumed as salads or in traditional remedies and contributebeneficiallyto human health [6,32,36]. Specifically, studies have demonstrated that obesity, diabetes or renal tubular injury induced by a high-fat diet caused a significant increase in the body weight, fasting blood glucose, serum TC and TG and uric acid levels in mice. Treatment with cichoric acid demonstrated positive effects on the above metabolic parameters by decreasing body weight, fasting blood glucose and serum TC and TG levels. Similarly, based on our work hereby, the anti-hyperlipidemic activity of *C. incana* indicates that such plants may play a key role as a rich source of cichoric acid with desirable effects on human health.

Nevertheless, despite the demonstrated promising results, it should be noted that plant extracts are exceedingly complex multicomponent mixtures that may influence biological processes by either playing an important role by acting synergetically, or else potentially negatively (due to antagonistic interactions of the molecules) or without any significant consequences. 

## 3. Materials and Methods

### 3.1. Plant Material

The plant material (aerial) parts of the selected plants were collected during the flowering stage and identified by Dr. E. Kalpoutzakis (Faculty of Pharmacy, National and Kapodistrian University of Athens) and Associate Prof. Th. Constantinidis (Faculty of Biology, National and Kapodistrian University of Athens). The voucher specimens were deposited at the Herbarium of Laboratory of Natural Products Chemistry, NKUA. The detailed information is presented in Table 7.

### 3.2. Extraction and Isolation

The air-dried aerial parts were finely ground and extracted at room temperature successively with cyclohexane (cHex): diethyl ether (Et_2_O): methanol (MeOH) [(1:1:1; 3 × 1 L, for 2 days each (non-polar extracts)] and MeOH: H_2_O [5:1; 3 × 1 L, for 1 day each (polar extracts)]. Amounts of 80–120 mg from the polar extract of each plant were extracted with n-butanol and the organic phase, after evaporation, was analyzed by LC-MS. The same residues were used for the NMR-metabolomic analysis. *C. incana* was selected for further analysis andpart of its MeOH: H_2_O (5:1) (9.0 g) extract was fractionated by VLC over silica gel (6.5 cm × 10.0 cm) using mixtures of increasing polarity (cHex: ethyl acetate: MeOH: H_2_O) with gradient elution and afforded ten fractions of 500 mL each (fractions RINA–RINJ) [5,7,8].

Vacuum liquid chromatography (VLC) was performed on a silica gel (Merck, Art. 7736). Fractionation was always monitored by TLC silica gel 60 F-254 (Merck, Art. 5554) and cellulose (Merck, Art. 5552) and visualized under UV (254 and 365 nm) and/or spraying with vanillin–sulfuric acid solution (Merck, Art. S26047 841) and with Neu’s reagent for phenolics (Alfa Aesar A16606). All obtained extracts and fractions were evaporated to dryness in a vacuum under low temperature and then placed in desiccators activated with P_2_O_5_ until their weights were stable.

### 3.3. Nuclear Magnetic Resonance (NMR) Spectroscopy

NMR spectra were measured in an AVANCE III 600 instrument equipped with a 5 mm TBI CryoProbe (^1^H-NMR 600 MHz, ^13^C-NMR 150 MHz) or a Bruker DRX 400 (^1^H-NMR 400 MHz, Bruker BioSpin) at 298 K. Chemical shifts are given in ppm (δ) and were referenced to the solvent signals at 3.31/49.0 ppm for methanol-d4 (CD_3_OD) and 7.24/77.0 ppm for CDCl_3_. COSY (correlation spectroscopy), HSQC (heteronuclear single-quantum correlation) and HMBC (heteronuclear multiple-bond correlation) experiments were performed using standard Bruker microprograms. 

During the whole analysis, all extracts and obtained subfractions were continuously monitored and traced down using an NMR metabolomic strategy, which permitted in-detail characterization of their chemical profiles. Furthermore, the 1D and 2D NMR spectra of the BuOH residues were measured (see SM) [15,39,40]. The metabolites were identified using NMR experiments, by comparing the obtained chemical shifts and coupling constant values with standards, as well as with the aid of LC-MS. All candidate structures were compared with those previously published in the literature.

### 3.4. Liquid Chromatography High-Resolution Quadrupole Time-of-Flight Mass Spectrometry (LC-Q-TOF-MS/MS)

Analyses of the butanol residues of each plant were performed with UHPLC Agilent 1290 infinity system with a DAD G4212A and MS Agilent G6540A Q-TOF with Agilent Jet Stream technology electrospray ionization. Separation was performed on a Phenomenex Luna Omega column (C18, 1.9 u, 90 A°, 75 × 2.0 mm) using gradient mixtures of 0.1% formic acid (solvent A) and MeCN supplemented with 0.1% formic acid (solvent B). Gradient: 0.0−8.0 min, 0→30% B; 8.0−8.1 min, 30→98% B; 8.1−9.1 min, 98% B; 9.1−9.2 min, 98→5% B; 9.2−10.0 min, 5% B; flow rate, 0.6 mL/min; injection volume, 1 μL; oven temperature, 40 °C. Data analysis was performed by MassHunter Workstation Software Qualitative Analysis (B.07.00, B.10.00, Agilent) using automatic mass spectrum integration. LC-Q-TOF-MS/MS analyses were performed in positive and negative ionization modes to obtain maximum information on its composition. The metabolites were characterized based on their mass spectra, using the precursor ion and comparison of the fragmentation patterns with molecules described in the literature [15]. The putative identification of these compounds is summarized in Table 1, where the compounds are listed according to their retention times in the total ion chromatogram (TIC).

### 3.5. In Vitro Activity

#### 3.5.1. DPPH (2,2-Diphenyl-1-Picrylhydrazyl) Radical Scavenging Assay

The free-radical scavenging capacity of *C. commutata*, *C. dioscoridis*, *C. foetida*, *C. heldreichiana*, *C. incana*, *C. rubra*, and *Phitosiacrocifolia* extracts and *Crepisincana* fractions RINA–RINKwere evaluated using the DPPH radicalsscavenging assay. Briefly, solid extracts dissolved in absolute methanol (at final concentrations of 0.01–0.5 mg/mL) were added to an equal volume of a methanolic solution of DPPH (Sigma-Aldrich) (final concentration 200 μM) at room temperature (22 ± 2 °C). The contents were mixed and incubated in the dark for 30 min and the absorbance measured at 517 nmby a Tecan Sunrise Microplate reader (Biodirect). In order to compare the radical scavenging efficiency of the extracts, the results were expressed as IC_50_ (mg/mL), i.e., the concentration that caused 50% scavenging of the DPPH radical [25,41].

#### 3.5.2. Total Phenolic Content-Folin–Ciocalteu(F-C) Protocol

Briefly, the same fractions as mentioned in paragraph 3.5.1 were used to quantifying the total phenolic content. Solid extracts were dissolved in methanol (1 mg/mL). F-C reagent (Sigma-Aldrich) was diluted 1:10 in water and was used together with a solution of sodium carbonate 7.5% *w/v* in water. Finally, solutions of gallic acid in methanol in various concentrations (0.02–0.1 mg/mL) were used for the reference curve. After 30 min of incubation, the absorbance (765 nm) was recorded by Tecan Infinite 200 Pro Microplate Reader using ELISA 96-wellplates. Each measurement was performed in triplicate [22,42].

### 3.6. In Vivo Activity

#### 3.6.1. Animals

Animal care was performed according to the guidelines established by the European Council Directive 2010/63/EU. The experimental procedures were approved by the National Peripheral Veterinary Authority Animal Ethics Committee after the affirmative opinion by the Animal Protocols Evaluation Committee. Male (hypolipidemic assay) and female (paw edema assay) C57BL/6 mice (3–6 months old) were used in this study. All mice originated from the breeding stock of the Small Animal Laboratory of the Section of Pharmaceutical Technology, Department of Pharmacy (EL 25 BIO 06). The animal’s room temperature and humidity were maintained at 24 ± 1 °C and 45%, respectively. The room was illuminated by yellow fluorescent tubes in a 12h cycle of light and dark (switched on at 8:00 and off at 20:00); these lamps do not emit any measurable UV radiation. The mice had unrestricted continuous access to standard solid pellets (Nuevo SA-Farma-Efyra Industrial & Commercial SA, Greece) and fresh water.

#### 3.6.2. Carrageenan-Induced Paw Edema Reduction Assay

For the in vivo anti-inflammatory activity, C57BL/6 female mice (6 per group, 5–6 months old, 20–34 g) were injected with 0.025 mL carrageenan (Merck KGaA, Darmstadt, Germany) (2% *w/v* solution in saline) i.d. into the right hind paw, with the left paw serving as the control. The test extracts *C. incana* 5:1, *C. heldreichiana* 5:1, *C. dioscoridis* 5:1 and fractions RINH and RINI, dissolved in saline with a few drops of Tween-80, were administered i.p. (0.3 mg/kg body weight) after the carrageenan injection. After 3.5 h, mice were sacrificed, hind legs were removed, and their weights measured in a precision analytical balance. The produced edema was estimated as the difference in weight (g) between the challenged right paw and the left paw. Results were expressed as the percentage of reduction in paw edema (mean from six animals per extract) [24,25,43].

#### 3.6.3. Hypolipidemic Activity Assay

For the in vivo hypolipidemic activity, C57BL/6 male mice (8 per group, 3–4 months old) were used. An aqueous solution of Triton WR 1339 (tyloxapol) was given i.p. to all mice (except the untreated group) at a dose of 30 mg/kg body weight and one hour later the test extracts (0.3 mg/kg body weight) of *C. incana* 5:1, *C. dioscoridis* 5:1 or fractions RINH and RINI, dissolved in saline or saline alone (for the control groups), were administered i.p. After 24 h, blood was drawn from the aorta/heart, the plasma was separated by centrifugation (15 min, 3000 rpm, 4 °C) and used for the determination of plasma total antioxidant capacity (TAC), total cholesterol (TC) and triglyceride (TG) levels, using commercially diagnostic kits [Biosis Biotechnological applications L.T.D., Athens, Greece]. Levels in plasma were determined in duplicate while the values presented are the mean from eight animals (per extract) [44,45]. For TAC, the average value of % reduction in DPPH which is considered 100% is 22.1%.

### 3.7. Statistical Analysis

All results are presented as means ± SD. The data were tested for normality of distribution. Data were evaluated by Student’s *t*-test. The *p*-value ≤ 0.05 was set as significance level for all data. Graphs were generated using GraphPad Prism 8.4.2 (GraphPad Software, Inc., San Diego, CA, USA).

## 4. Conclusions

*Crepis* is a widely distributed genus that can be found in different types of habitats around the globe, with the Mediterranean area being the most important center of its diversification. This study investigated the antioxidant, anti-inflammatory and anti-hyperlipidemic properties of *Crepis* spp. According to our findings, *Crepis* spp. is a rich source of phenolic compounds and, specifically, polyphenolic and flavonoid derivatives. In particular, the *Crepis* species may play a key role as an alternative source of caffeoyl tartaric acid derivatives, such as cichoric and caftaric acid. These promising secondary metabolites attract the interest of the scientific community due to their numerous biological activities, including antioxidant, anti-diabetic and anti-inflammatory potential. The specific pharmacological profile exhibited by the studied hereby *C. incana* extract justifies its further investigation and renders it attractive for use as a potential beneficial nutritional supplement. However, other important aspects of the genus, such as the analysis of new promising bioactivities, including the elucidation of their mechanisms of action and potential toxicity in higher doses, should be further investigated.

## Figures and Tables

**Table 1:** LC-MS analyses on *Crepis* spp.

	Positive Ion Mode	Negative Ion Mode				*C. dioscoridis*	*C. incana*	*C. heldreichiana*	*C. foetida*	*C. commutata*	*C. rubra*	*P. crocifolia*
Rt	Found	Found	Mass	Molecular Formula	Proposed Compounds							
0.324	203.0530 [M+Na]^+^	179.0563 [M-H]^−^	180.0635	C_6_H_12_O_6_	hexose	•	•	•	•	•	•	•
0.337	365.1059 [M+Na]^+^	341.1093 [M-H]^−^	342.1163	C_12_H_22_O_11_	carbohydrates	•	•	•	•	•	•	•
0.490	349.1121 [M+Na]^+^	371.1189 [M+HCOO]^−^	326.1217	C_12_H_22_O_10_	carbohydrates	•	•	•	•	•	•	•
2.046	181.0498 [M+H]^+^	179.0350 [M-H]^−^	180.0421	C_9_H_8_O_4_	caffeic acid isomer	•	•	•	•	•	•	•
2.502		311.0407 [M-H]^−^	312.0478	C_13_H_12_O_9_	caftaric acid	•	•	•	•	•	•	
2.610	309.0947 [M+Na]^+^	331.1035 [M+HCOO]^−^	286.1049	C_13_H_18_O_7_	salicin isomer	•			•		•	
2.997	309.0947 [M+Na]^+^	331.1035 [M+HCOO]^−^	286.1045	C_13_H_18_O_7_	salicin isomer	•			•		•	
3.005	343.1026 [M+H]^+^		342.0952	C_15_H_18_O_9_	caffeic acid glycoside	•	•	•	•	•	•	•
3.050	341.0869 [M+H]^+^	339.0719 [M-H]^−^	340.0796	C_15_H_16_O_9_	cichoriin	•	•	•	•	•	•	•
3.210	153.0546 [M+H]^+^	151.0400 [M-H]^−^	152.0468	C_8_H_8_O_3_	methoxybenzoic acid	•	•		•	•	•	
3.483	293.0993 [M+Na]^+^	315.1084 [M-HCOO]^−^	270.1103	C_13_H_18_O_6_	benzyl glycoside	•	•	•	•	•	•	•
3.604	355.1028 [M+H]^+^	353.0888 [M-H]^−^	354.0957	C_16_H_18_O_9_	caffeoylquinic acid isomer	•	•		•	•	•	
3.629	355.1028 [M+H]^+^	353.0879 [M-H]^−^	354.0953	C_16_H_18_O_9_	caffeoylquinic acid isomer	•	•	•	•	•	•	•
3.772	181.0496 [M+H]^+^	179.0349 [M-H]^−^	180.0421	C_9_H_8_O_4_	caffeic acid isomer	•	•	•	•	•	•	•
4.246	337.0893 [M+H]^+^		336.0820	C_16_H_16_O_8_	caffeoylshikimic acid isomer	•						
4.509	337.0917 [M+H]^+^	335.077 [M-H]^−^	336.0844	C_16_H_16_O_8_	caffeoylshikimic acid isomer	•		•				
4.529	449.1783 [M+Na]^+^	471.1868 [M+HCOO]^−^	426.2891	C_21_H_30_O_9_	sesquiterpene lactone glycoside							•
4.530	449.1783 [M+Na]^+^	471.1868 [M+HCOO]^−^	426.1890	C_21_H_30_O_9_	sesquiterpene lactone glycoside	•	•	•	•	•	•	
4.580	449.1780 [M+Na]^+^	471.1868 [M+HCOO]^−^	426.1897	C_21_H_30_O_9_	sesquiterpene lactone glycoside			•	•	•		
4.707	165.0548 [M+H]^+^	163.0398 [M-H]^−^	164.0473	C_9_H_8_O_3_	coumaric acid	•	•	•	•	•	•	
4.790	611.1607 [M+H]^+^	609.1458 [M-H]^−^	610.1535	C_27_H_30_O_16_	luteolin diglycoside isomer							
4.997	449.1082 [M+H]^+^	447.0937 [M-H]^−^	448.1009	C_21_H_20_O_11_	luteolin glycoside isomer	•	•	•	•		•	
5.151	465.1027 [M+H]^+^	463.0885 [M-H]^−^	464.0954	C_21_H_20_O_12_	quercetin glycoside isomer					•		
5.199	497.0688 [M+Na]^+^	473.0732 [M-H]^−^	474.0794	C_22_H_18_O_12_	cichoric acid isomer	•	•	•	•	•	•	•
5.216	611.1607 [M+H]^+^	609.1458 [M-H]^−^	610.153	C_27_H_30_O_16_	luteolin diglycoside isomer					•		
5.363	447.1617 [M+Na]^+^	469.1715 [M+HCOO]^−^	424.1733	C_21_H_28_O_9_	sesquiterpene lactone glycoside	•	•			•	•	
5.571	611.1609 [M+H]^+^	609.1458 [M-H]^−^	610.1534	C_27_H_30_O_16_	luteolin diglycoside isomer	•	•	•	•	•	•	•
5.716	463.087 [M+H]^+^	461.0724 [M-H]^−^	462.0798	C_21_H_18_O_12_	luteolin glucuronide	•	•	•	•	•	•	•
5.733	465.1029 [M+H]^+^	463.0885 [M-H]^−^	464.0954	C_21_H_20_O_12_	quercetin glycoside isomer	•	•	•	•	•	•	
5.751		285.0406 [M-H]^−^	286.0477	C_15_H_10_O_6_	luteolin isomer	•	•	•	•	•	•	
5.760	449.1081 [M+H]^+^	447.0939 [M-H]^−^	448.101	C_21_H_20_O_11_	luteolin glycoside isomer	•	•	•	•	•	•	•
5.780	409.1859 [M+H]^+^		408.1784	C_21_H_28_O_8_	sesquiterpene lactone glycoside							•
5.890	409.1830 [M+H]^+^		408.1759	C_21_H_28_O_8_	sesquiterpene lactone glycoside			•				
5.930	517.1347 [M+H]^+^	515.1193 [M-H]^−^	516.1270	C_25_H_24_O_12_	dicaffeoylquinic acid isomer	•	•	•	•	•	•	•
5.959	265.1436 [M+H]^+^		264.1363	C_15_H_20_O_4_	sesquiterpene lactone	•	•		•	•	•	
6.021	465.1029 [M+H]^+^	463.0880 [M-H]^−^	464.0957	C_21_H_20_O_12_	luteolin glycoside isomer	•				•	•	
6.029	551.1033 [M+H]^+^	549.0887 [M-H]^−^	550.0960	C_24_H_22_O_15_	quercetin malonylglycoside isomer	•	•	•	•	•	•	
6.116		609.1454 [M-H]^−^	610.1528	C_27_H_30_O_16_	luteolin diglycoside isomer	•			•	•	•	
6.160	517.1347 [M+H]^+^	515.1195 [M-H]^−^	516.1273	C_25_H_24_O_12_	dicaffeoylquinic acid acid isomer	•	•	•	•	•		•
6.244	195.0650 [M+H]^+^	193.0505 [M-H]^−^	194.0578	C_10_H_10_O_4_	methyl caffeate	•	•	•	•		•	•
6.253	625.1764 [M+H]^+^	623.1615 [M-H]^−^	624.1791	C_28_H_32_O_16_	isorhamnetin-rutinoside isomer	•	•	•	•	•	•	
6.289	287.0549 [M+H]^+^	285.0406 [M-H]^−^	286.0477	C_15_H_10_O_6_	luteolin isomer	•		•				
6.296	449.1081 [M+H]^+^	447.0935 [M-H]^−^	448.1009	C_21_H_20_O_11_	luteolin glycoside isomer	•	•	•	•			
6.316		371.1342 [M+HCOO]^−^	372.1422	C_16_H_22_O_7_	eugenyl glycoside isomer	•	•	•		•	•	
6.505	517.1343 [M+H]^+^	515.1193 [M-H]^−^	516.1268	C_25_H_24_O_12_	dicaffeoylquinic acid isomer	•	•	•	•	•	•	•
6.436	433.1134 [M+H]^+^	431.0981 [M-H]^−^	432.1993	C_21_H_20_O_10_	apigenin glycoside	•	•	•	•	•	•	•
7.130	449.1783 [M+Na]^+^	471.1868 [M+HCOO]^−^	426.2890	C_21_H_30_O_9_	sesquiterpene lactone glycoside		•					
7.240	449.1783 [M+Na]^+^	471.1868 [M+HCOO]^−^	426.2891	C_21_H_30_O_9_	sesquiterpene lactone glycoside	•	•					
7.381	492.1134 [M+NH_4_]^+^	473.0724 [M-H]^−^	474.2099	C_22_H_18_O_12_	cichoric acid isomer	•	•	•			•	
7.813		301.0349 [M-H]^−^	302.0422	C_15_H_10_O_7_	quercetin isomer	•	•					
7.876	287.0548 [M+H]^+^	285.0405 [M-H]^−^	286.0478	C_15_H_10_O_6_	luteolin isomer	•	•	•	•	•	•	•

Shadedsquares (**•**) indicates presence of metabolites.

**Table 2:** Total polyphenol content (TPC) of plant extracts, fractions and reference compounds, expressed as TPC or GAE values (F-C assay).

Extract (1 mg/mL)	TPC (mg/mL) or GAE (GAE/gEx) ^a^	Fractions (1 mg/mL)	TPC (mg/mL) or GAE (GAE/gEx) ^a^
*C. commutata* 5:1	0.0488	RINA	0
*C. dioscoridis* 5:1	0.0561	RINB	0.1282
*C. foetida* 5:1	0.0499	RINC	0.1712
*C. foetida* H_2_O	0.0518	RIND	0.0796
*C. heldreichiana* 5:1	0.0521	RINE	0.0227
*C. incana* 5:1	0.0834	RINF	0.0937
*C. rubra* 5:1	0.0564	RINH	0.0830
*C. rubra* H_2_O	0.0414	RINI	0.1210
*P. crocifolia* 5:1	0.0515	RINJ	0.0814

^a^ TPC = total phenolic content, GAE = gallic acid equivalents.

**Table 3:** Antioxidant effect of plant extracts, fractions and reference compounds, expressed as IC_50_ values (DPPH assay).

Extracts	IC_50_ (mg/mL)	Fractions	IC_50_ (mg/mL)	Reference	IC_50_ (mg/mL)
*C. commutata* 5:1	0.14	RINA	>1	RSV	0.02
*C. dioscoridis* 5:1	0.12	RINB	0.14	ASC	0.01
*C. foetida* 5:1	0.14	RINC	0.24	BHT	0.38
*C. foetida* H_2_O	>1	RIND	0.12		
*C. heldreichiana* 5:1	0.10	RINE	0.35		
*C. incana* 5:1	0.07	RINF	>1		
*C. rubra* 5:1	0.19	RINH	0.12		
*C. rubra* H_2_O	0.30	RINI	0.06		
*P. crocifolia* 5:1	0.26	RINJ	0.11		
		RINK	>1		

**Table 4:** Effect of extract *C. incana* 5:1, *C. dioscoridis* 5:1 and fractions RINH and RINI on TAC.

Groups	TAC ^a^	% TAC Increase (vs. Control)	% TAC Decrease (vs. Untreated)
Untreated	100% (±11.8) ***	-	-
Control	63.3% (±12.2)	-	36.7
*C. incana* 5:1	94.1% (±12.2) ***	48.7	5.9
*C. dioscoridis* 5:1	84.6% (±7.1) * ^#^	33.6	15.4
RINH	83.7% (±5.4) ** ^##^	32.2	16.3
RINI	71.9% (±7.7) ^###^	13.6	28.1

^a^ Total antioxidant capacity (TAC) is measured as % reduction of a solution of DPPH. The TAC value of the Untreated group is considered 100%. The effect of other groups (extracts/fractions) is expressed as a percentage of TAC increase compared to Control and TAC decrease compared to Untreated. Each value represents the mean ± SD obtained from eightanimals. Significant difference of Control group from Untreated group: *** *p* = 0.0001, *C. incana* 5:1 from Control group: *** *p* = 0.0001, *C*. *dioscoridis* from Control group: * *p* = 0.0004 and from Untreated group: # *p* = 0.0035, and RINH from Control group: ** *p* = 0.0003 and from Untreated group: ## *p* = 0.0016, RINI from Untreated group: ### *p* = 0.0001.

**Table 5:** Effect of extract *C. incana* 5:1, *C. dioscoridis* 5:1 and fractions RINH, RINI on total cholesterol plasma levels (TC).

Groups	TC (mg/dL)	% TC Decrease (vs. Control)	% TC Increase (vs. Untreated)
Untreated	96.4 (±6.7) *	-	-
Control	124.0 (±12.7)	-	28.6
*C. incana* 5:1	58.4 (±8.9) ** ^##^	52.9	−39.4
*C. dioscoridis* 5:1	53.0 (±16.2)	57.3	−45.0
RINH	77.2(±16.3)	37.7	−19.9
RINI	74.3(±7.1)	40.1	−22.9

The effect is expressed as a percentage of reduction in total cholesterol levels in plasma, compared to Control and Untreated. Each value represents the mean ± SD obtained from eight animals. Significant difference of Control from Untreated group: * *p* = 0.045. *C. incana* 5:1 from Control: ** *p* = 0.001, from Untreated group: ## *p* = 0.0045.

**Table 6:** Effect of extract *C. incana* 5:1, *C. dioscoridis* 5:1 and fractions RINH, RINI on triglyceride plasma levels (TG).

Groups	TG (mg/dL)	% TG Decrease (vs. Control)	% TG Increase (vs. Untreated)
Untreated	59.0 (±6.1)	-	-
Control	123.5 (±37.9)	-	109.3
*C. incana* 5:1	77.5 (±15.1)	37.2	31.4
*C. dioscoridis* 5:1	110.4 (±11.5) *	10.6	87.1
RINH	99.5 (±12.7)	19.4	68.6
RINI	89.0 (±22.7)	27.9	50.8

The effect is expressed as a percentage of reduction in total triglyceride levels in plasma, compared to Control and Untreated group. Each value represents the mean ± SD obtained from eight animals. Significant difference of *C. dioscoridis* 5:1 from Control group: * *p* = 0.02.

**Table 7:** Collection data of the investigated *Crepis* spp.

Taxon	Locality	Latitude	Longitude	Altitude	Date	Voucher
*C. commutata* (Spreng.) Greuter	Anabyssos	37°43″ N	23°55″ E	1400–2400 m	4-2016	Skaltsaand Barda 001
*C. dioscoridis* L.	Boumistos mountain	38°70657′ N	21°04338′ E	487 m	6-2016	Skaltsaand Tsoukalas 001
*C. foetida* L.	Phaleron	37°56′23.24″ Ν	23°40′ 55.83″ Ε	4 m	5-2019 6-2016	Kalpoutzakis5122/5-5-2019
*C. heldreichiana* (Kuntze) Greuter	Taygetos mountain	36°56’43.3″ N	22°21’16.4″ E	1450–2300 m	6-2017	Skaltsaand Grafakou 004
*C. incana* Sm.	Dirphys mountain	38°61″ N	23°85″ E	1100–1200 m	6-2012	Skaltsaand Tsoukalas 002
*C. rubra* L.	Cithaeron mountain	38°10′11.25″ Ν	23°18′31.75″ Ε	647 m	6-10-2018	Kalpoutzakis5115/2-5-2019
*Phitosiacrocifolia* Kamari andGreuter	Parnonas mountain	37°16.897′ Ν	22°36.760′ Ε	1860 m	6-10-2018	Kalpoutzakis5036/6-10-2018

## Data Availability

Not applicable.

## Supplementary Materials

The following supporting information can be downloaded at: https://www.mdpi.com/article/10.3390/molecules27196173/s1, S1.1–S1.8:LC-MS chromatograms; S2.1–S2.5: MS-MS spectrum of selected compounds; S3.1–S3.28: NMR data.
