# Supplementary material for: A Bio-Guided Screening for Antioxidant, Anti-Inflammatory and Hypolipidemic Potential Supported by Non-Targeted Metabolomic Analysis of Crepis spp
Source: Molecules. 2022 Sep 20;27(19):6173. doi: 10.3390/molecules27196173 (PMC9571268; doi:10.3390/molecules27196173)

A Bio-Guided Screening for Antioxidant, Anti-Inflammatory and Hypolipidemic Potential Supported by Non-Targeted Metabolomic Analysis of *Crepis* spp.

Christina Barda [1,2], Konstantina Anastasiou [3], Ariadni Tzara [3], Maria-Eleni Grafakou [1], Eleftherios Kalpoutzakis [1], Joerg Heilmann [2], Michael Rallis [4,] Angeliki P. Kourounakis [3,*] and Helen Skaltsa [1,*]

[1] Department of Pharmacognosy & Chemistry of Natural Products, Faculty of Pharmacy, School of Health Sciences, National & Kapodistrian University of Athens, 15771 Athens, Greece

[2] Department of Pharmaceutical Biology, Faculty of Pharmacy and Chemistry, University of Regensburg, D-93053 Regensburg, Germany

[3] Department of Medicinal Chemistry, Faculty of Pharmacy, School of Health Sciences, National & Ka-podistrian University of Athens, 15771 Athens, Greece

[4] Unity of Dermatopharmacology, Department of Pharmaceutical Technology, Faculty of Pharmacy, School of Health Sciences, National & Kapodistrian University of Athens, 15771 Athens, Greece

* Correspondence: angeliki@pharm.uoa.gr (A.P.K.); skaltsa@pharm.uoa.gr (H.S.); Tel.: +30-697-438-8878 (A.P.K.); +30-697-262-3095 (H.S.)

Keywords: *Crepis*; Asteraceae; LC-MS; NMR; cichoric acid; phenolic acid; biological activity; mouse paw edema; antihyperlipidemic

Supplementary Materials

S1. LC-MS 4
S1.1 LC-ESI-MS of BuOH extract of *Crepis dioscoridis* in positive (A) and negative ion (B) modes. 4
S1.2 LC-ESI-MS of BuOH extract of *Crepis sibthorpiana* in positive (A) and negative ion (B) modes. ... 6
S1.3 LC-ESI-MS of BuOH extract of *Crepis heldreichiana* in positive (A) and negative ion (B) modes... 8
S1.4 LC-ESI-MS of BuOH extract of *Crepis commutata* in positive (A) and negative ion (B) modes. 10
S1.5 LC-ESI-MS of BuOH extract of *Phitosia crocifolia* in positive (A) and negative ion (B) modes..... 12
S1.6 LC-ESI-MS of BuOH extract of *Crepis rubra* in positive (A) and negative ion (B) modes. 14
S1.7 LC-ESI-MS of BuOH extract of *Crepis foetida* in positive (A) and negative ion (B) modes. 16
S1.8 LC-ESI-MS of BuOH extract of *Crepis incana* in positive (A) and negative ion (B) modes............ 18
S.2 MS-MS spectrum of selected compounds in positive (A) and/or negative (B) ion mode 20
S.2.1 MS-MS spectrum of cichoric acid.. 20
S.2.2 MS-MS spectrum of chlorogenic acid isomer.. 21
S.2.3 MS-MS spectrum of luteolin 7-glucoside (Cynaroside) 22
S.2.4 MS-MS spectrum of Di-caffeoylquinic acid isomer.. 23
S.2.5 MS-MS spectrum of luteolin.. 23
S3. NMR data 25
S3.1 ^{1}H NMR (600 MHz, CD_3OD) spectrum of *Crepis rubra* 25
S3.2 ^{13}C NMR (150 MHz, CD_3OD) spectrum of *C. rubra* 25
S3.3 HSQC (CD_3OD) spectrum of *C. rubra* 26
S3.4 HMBC (CD_3OD) spectrum of *C. rubra* 27
S3.5 ^{1}H NMR (600 MHz, CD_3OD) spectrum of *Crepis dioscoridis* 27
S3.6 ^{13}C NMR (150 MHz, CD_3OD) spectrum of *C. dioscoridis* 28
S3.7 HSQC (CD_3OD) spectrum of *C. dioscoridis* 28
S3.8 HMBC (CD_3OD) spectrum of *C. dioscoridis* 29
S3.9 ^{1}H NMR (600 MHz, CD_3OD) spectrum of *Crepis foetida* 29
S3.10 ^{13}C (150 MHz, CD_3OD) spectrum of *C. foetida* 30
S3.11 HSQC (CD_3OD) spectrum of *C. foetida* 30
S3.12. HMBC (CD_3OD) spectrum of *C. foetida* 31
S3.13 ^{1}H NMR (600 MHz, CD_3OD) spectrum of *Crepis commutata* 31

S3.14 ^{13}C NMR (150 MHz, CD_3OD) spectrum of *C. commutata* 32
S3.15 HSQC (CD_3OD) spectrum of C. *commutata* 32
S3.16 HMBC (CD_3OD) spectrum of *C.commutata* 33
S3.17 ^{1}H NMR (600 MHz, CD_3OD) spectrum of *Phitosia crocifolia* 33
S3.18 ^{13}C NMR (150 MHz, CD_3OD) spectrum of *P. crocifolia* 34
S3.19 HSQC (CD_3OD) spectrum of *P. crocifolia* 34
S3.20 HMBC (CD_3OD) spectrum of *P. crocifolia* 34
S3.21 ^{1}H NMR (600 MHz, CD_3OD) spectrum of *Crepis incana* 35
S3.22 ^{13}C NMR (150 MHz, CD_3OD) spectrum of *C. incana* 36
S3.23 HSQC (CD_3OD) spectrum of *C. incana* 36
S3.24 HMBC (CD_3OD) spectrum of *C. incana* 37
S3.25 ^{1}H NMR (600 MHz, CD_3OD) spectrum of *Crepis heldreichiana* 37
S3.26 ^{13}C NMR (150 MHz, CD_3OD) spectrum of *C. heldreichiana* 38
S3.27 HSQC (CD_3OD) spectrum of *C. heldreichiana* 38
S3.28 HMBC (CD_3OD) spectrum of *C. heldreichiana* 39

S1. LC-MS

S1.1 LC-ESI-MS of BuOH extract of *Crepis dioscoridis* in positive (A) and negative ion (B) modes.

A. LC-MS ESI+

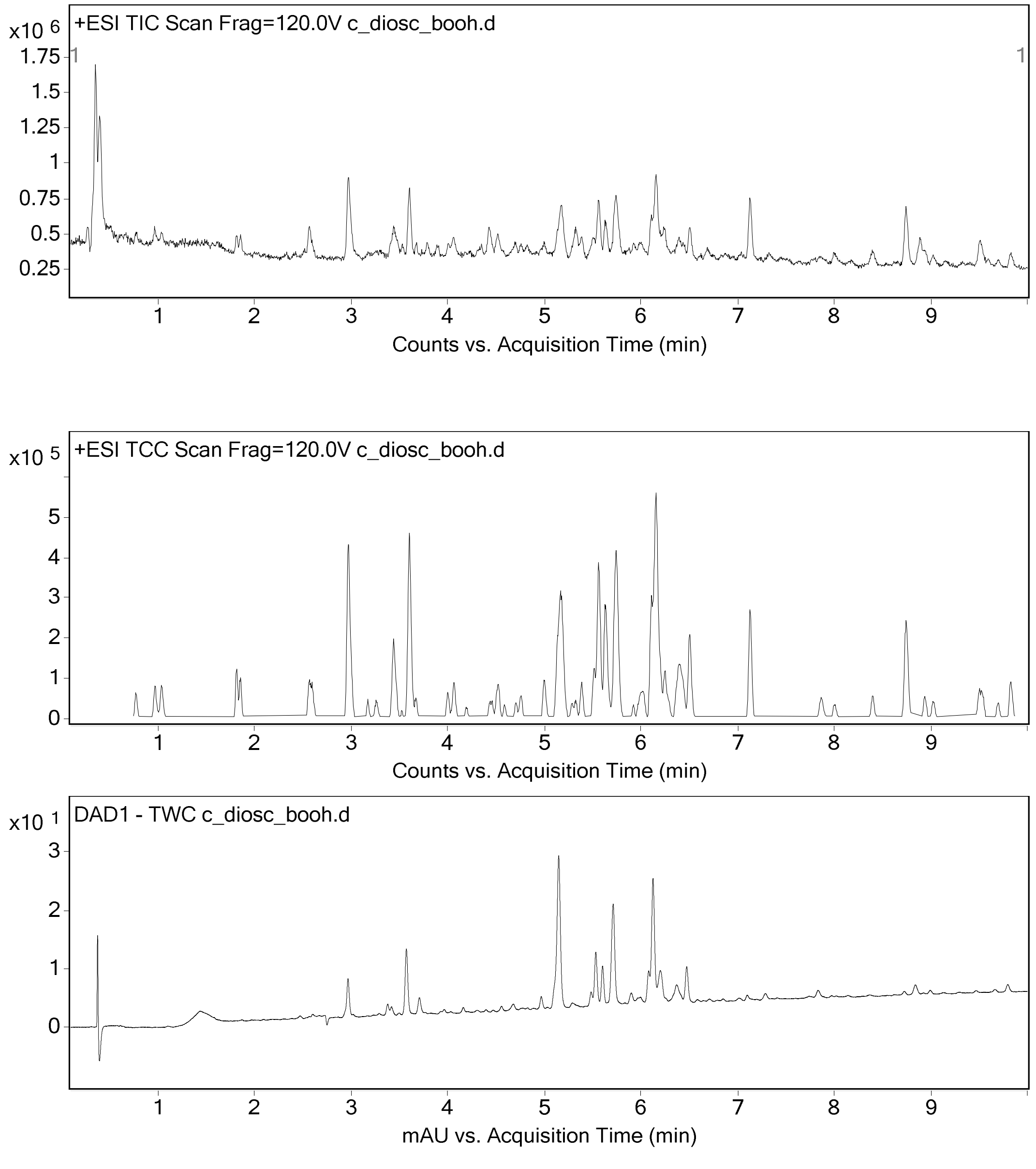

B. LC-MS ESI-

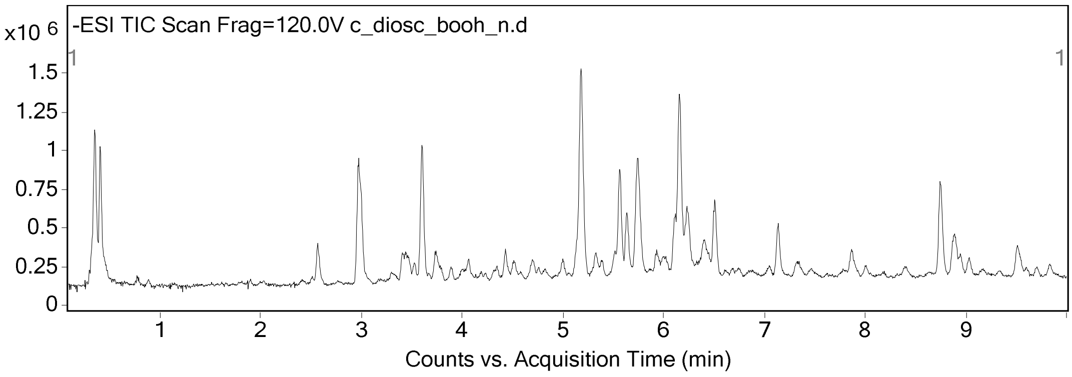
-ESI TIC Scan Frag=120.0V c_diosc_booh_n.d
x10 6
1.5
1.25
1
0.75
0.5
0.25
0
1 2 3 4 5 6 7 8 9
Counts vs. Acquisition Time (min)

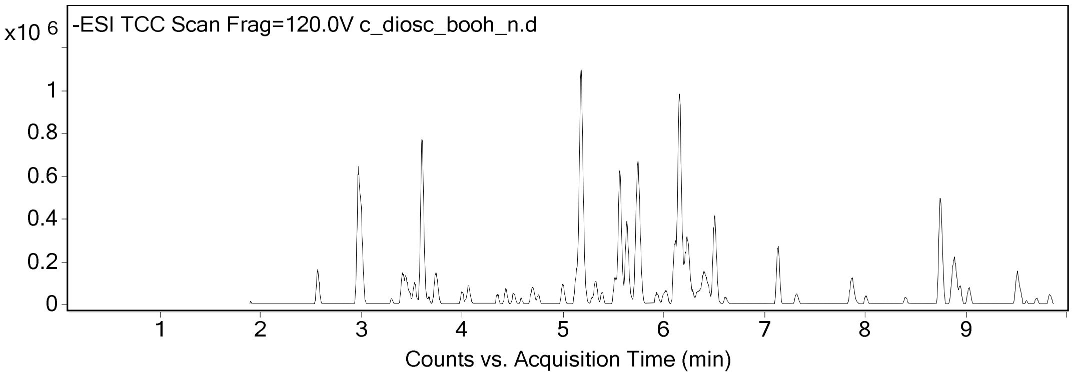
-ESI TCC Scan Frag=120.0V c_diosc_booh_n.d
x10 6
1
0.8
0.6
0.4
0.2
0
1 2 3 4 5 6 7 8 9
Counts vs. Acquisition Time (min)

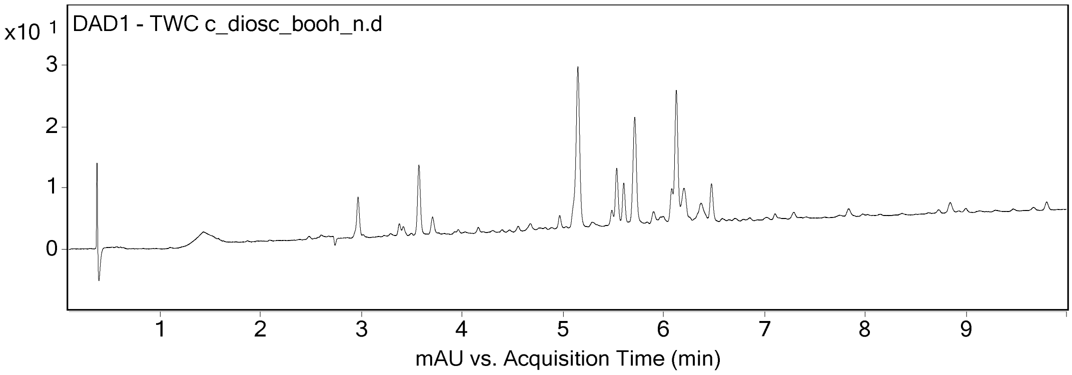
DAD1 - TWC c_diosc_booh_n.d
x10 1
3
2
1
0
1 2 3 4 5 6 7 8 9
mAU vs. Acquisition Time (min)

S1.2 LC-ESI-MS of BuOH extract of *Crepis sibthorpiana* in positive (A) and negative ion (B) modes.

A. LC-MS ESI+

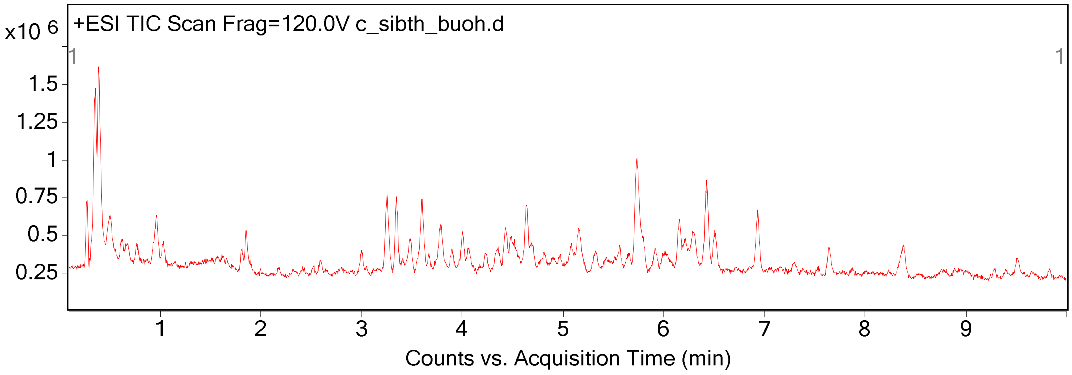

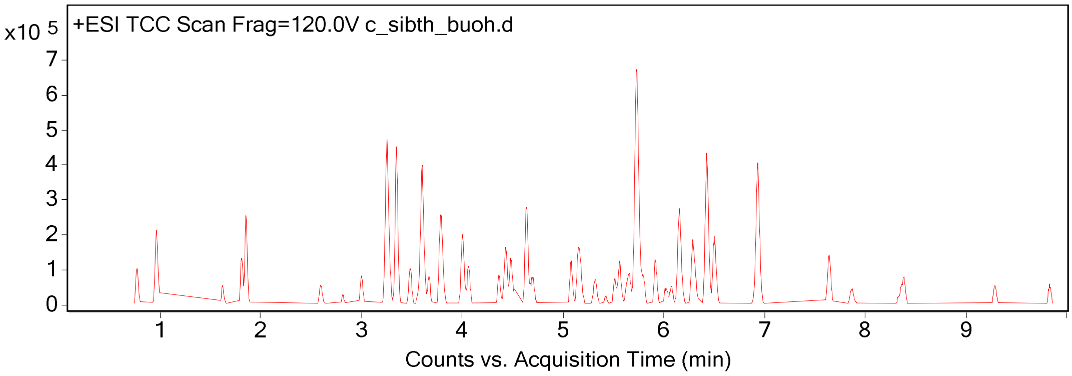

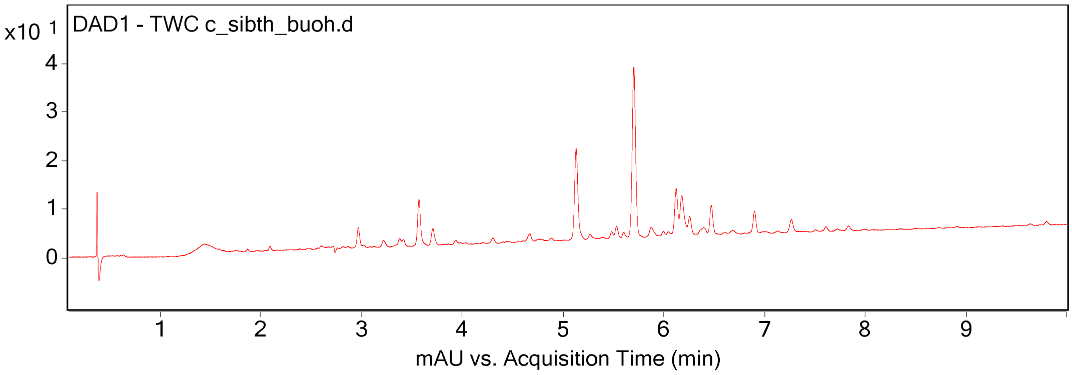

B. LC-MS ESI-

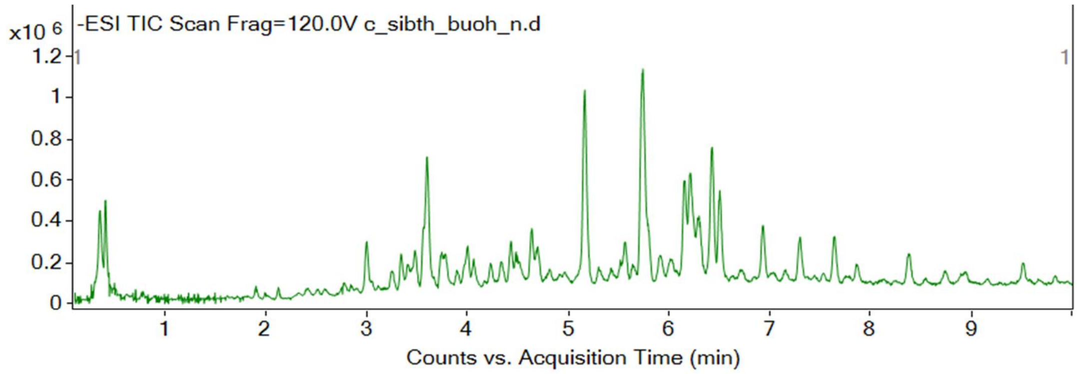

-ESI TIC Scan Frag=120.0V c_sibth_buoh_n.d
x10 6
Counts vs. Acquisition Time (min)

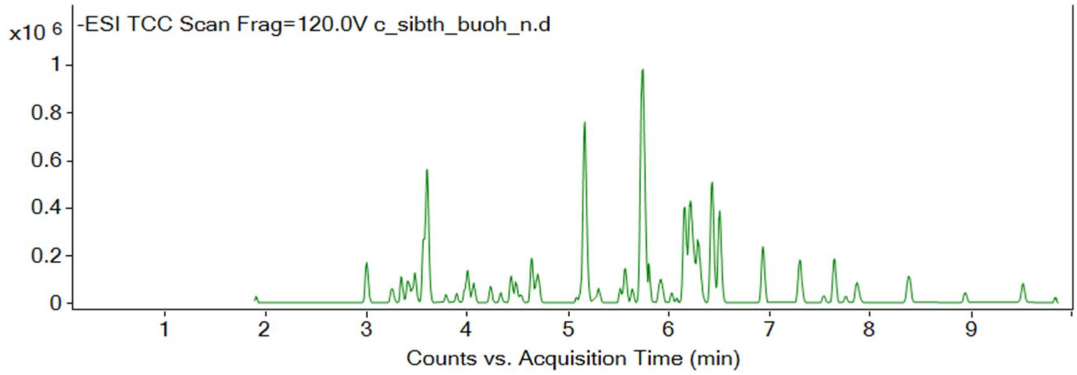

-ESI TCC Scan Frag=120.0V c_sibth_buoh_n.d
x10 6
Counts vs. Acquisition Time (min)

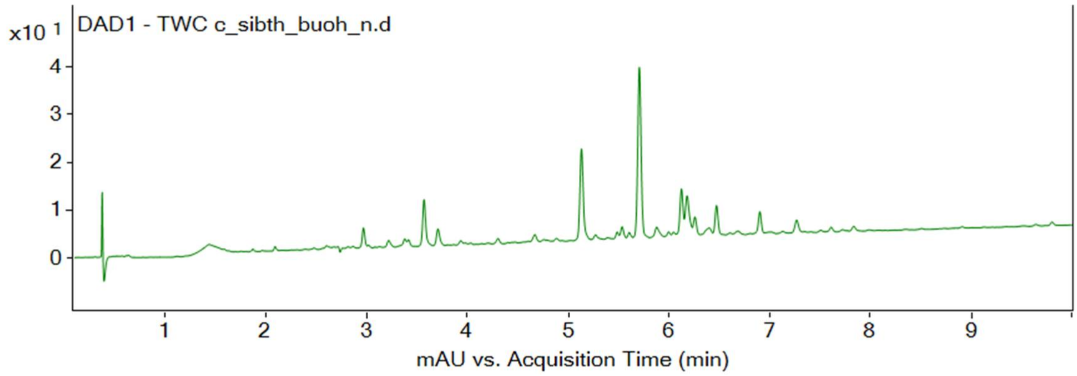

DAD1 - TWC c_sibth_buoh_n.d
x10 1
mAU vs. Acquisition Time (min)

S1.3 LC-ESI-MS of BuOH extract of *Crepis heldreichiana* in positive (A) and negative ion (B) modes.

A. LC-MS ESI+

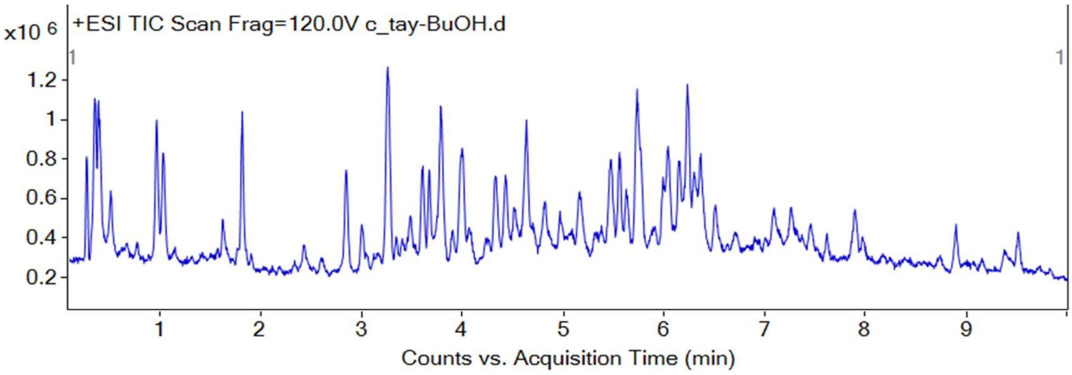

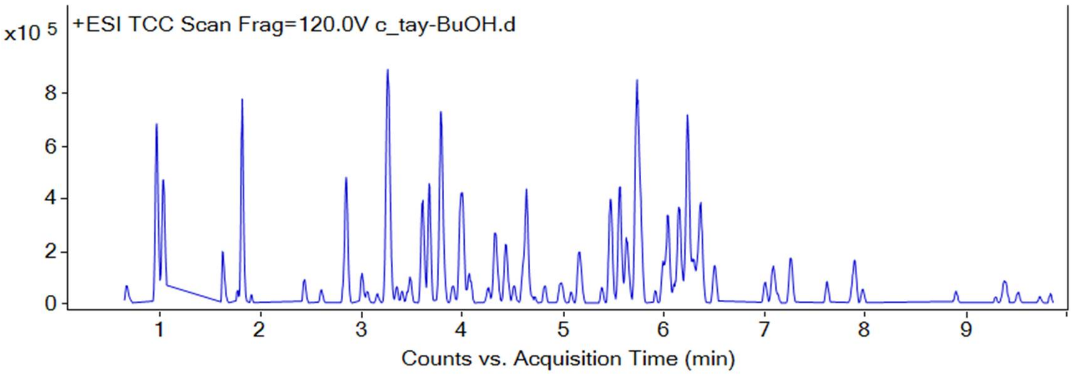

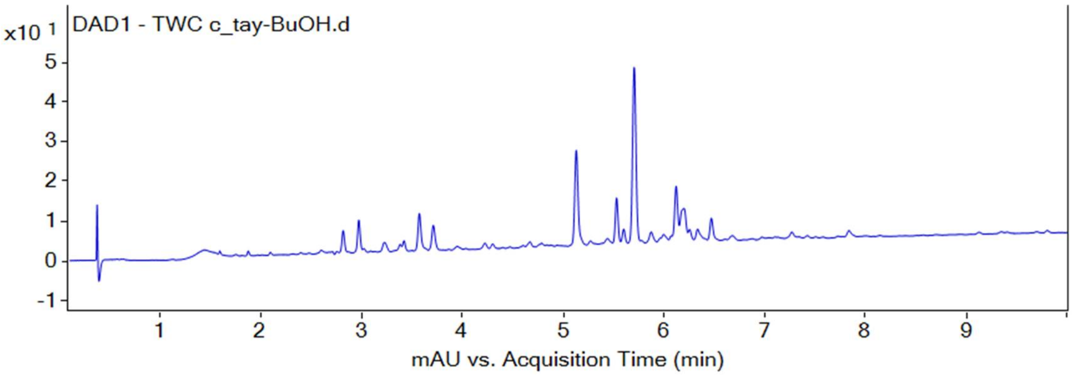

B. LC-MS ESI-

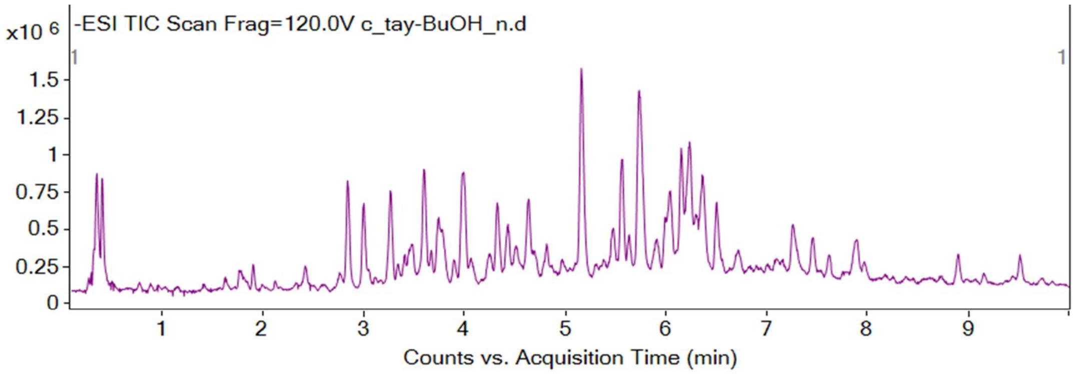
x10 6
-ESI TIC Scan Frag=120.0V c_tay-BuOH_n.d
1.5
1.25
1
0.75
0.5
0.25
0
1 2 3 4 5 6 7 8 9
Counts vs. Acquisition Time (min)

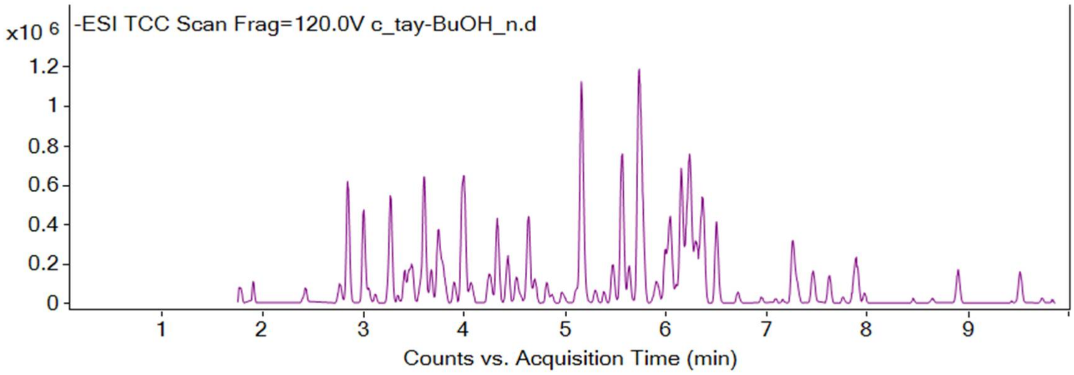
x10 6
-ESI TCC Scan Frag=120.0V c_tay-BuOH_n.d
1.2
1
0.8
0.6
0.4
0.2
0
1 2 3 4 5 6 7 8 9
Counts vs. Acquisition Time (min)

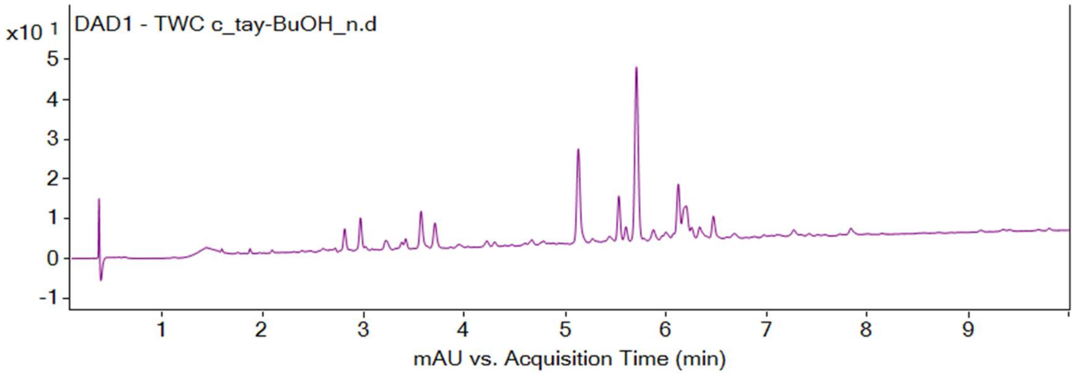
x10 1
DAD1 - TWC c_tay-BuOH_n.d
5
4
3
2
1
0
-1
1 2 3 4 5 6 7 8 9
mAU vs. Acquisition Time (min)

S1.4 LC-ESI-MS of BuOH extract of *Crepis commutata* in positive (A) and negative ion (B) modes.

A. LC-MS ESI+

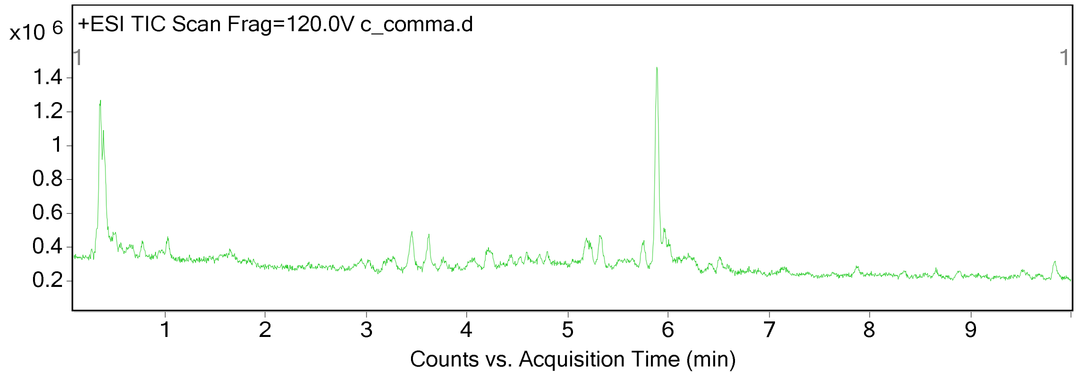

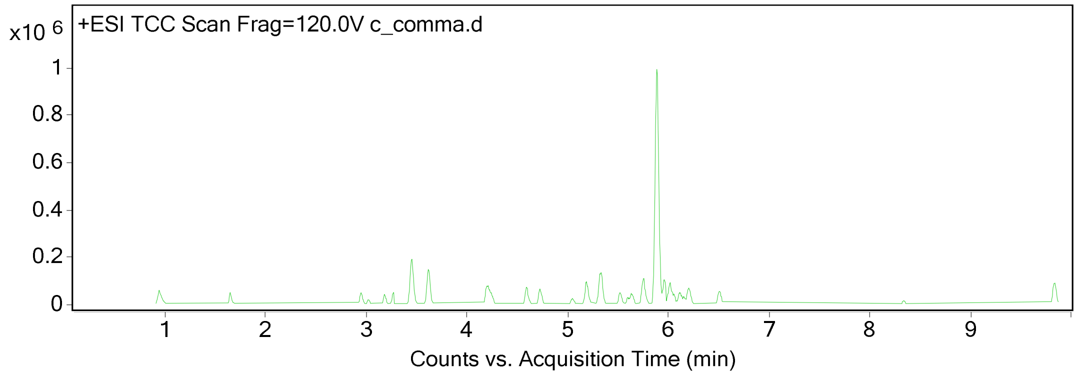

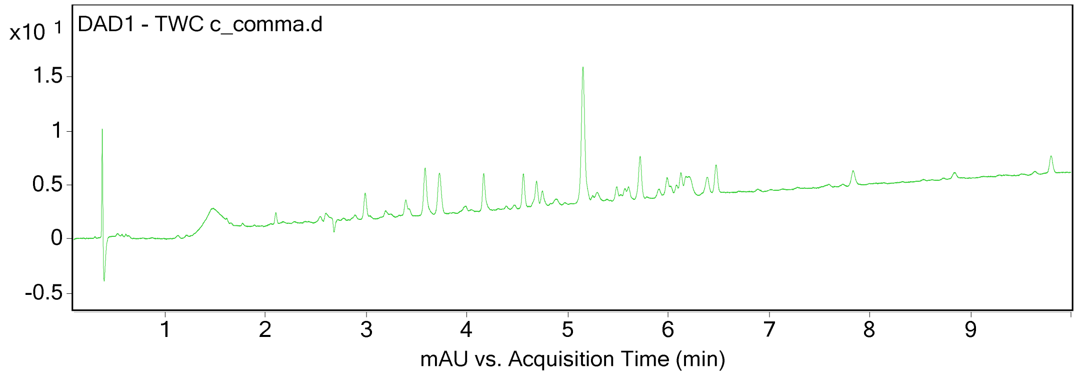

B. LC-MS ESI-

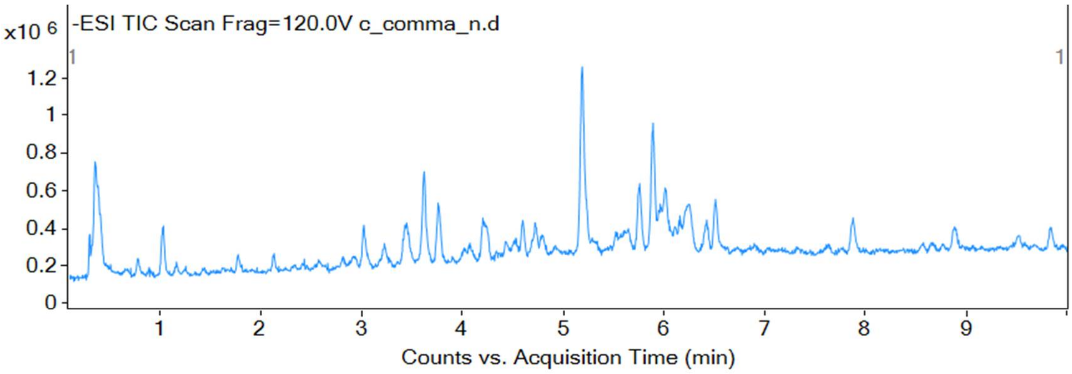
x10 6
-ESI TIC Scan Frag=120.0V c_comma_n.d
1.2
1
0.8
0.6
0.4
0.2
0
1 2 3 4 5 6 7 8 9
Counts vs. Acquisition Time (min)

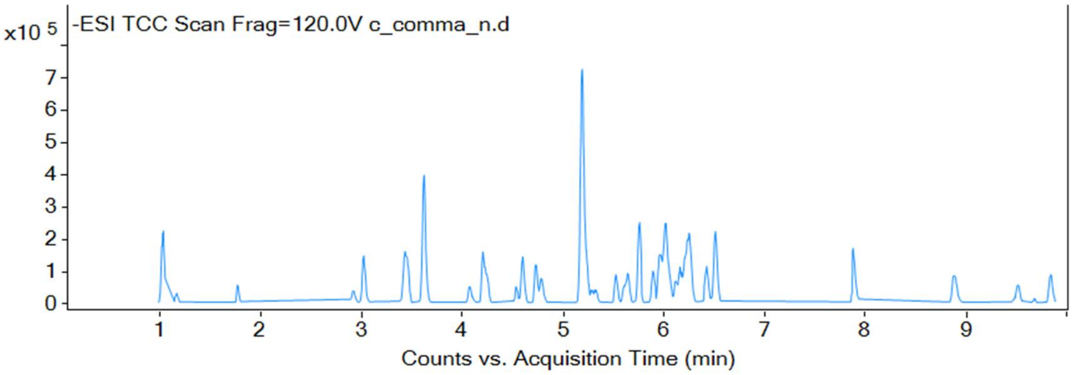
x10 5
-ESI TCC Scan Frag=120.0V c_comma_n.d
7
6
5
4
3
2
1
0
1 2 3 4 5 6 7 8 9
Counts vs. Acquisition Time (min)

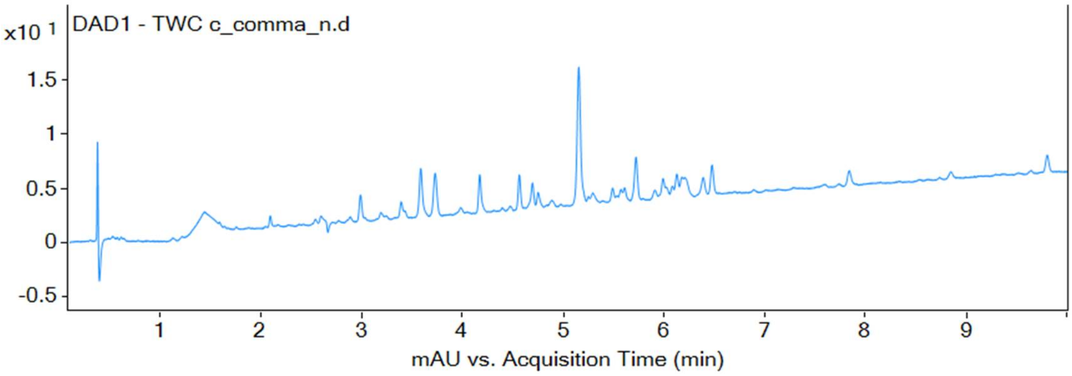
x10 1
DAD1 - TWC c_comma_n.d
1.5
1
0.5
0
-0.5
1 2 3 4 5 6 7 8 9
mAU vs. Acquisition Time (min)

S1.5 LC-ESI-MS of BuOH extract of *Phitosia crocifolia* in positive (A) and negative ion (B) modes.

A. LC-MS ESI+

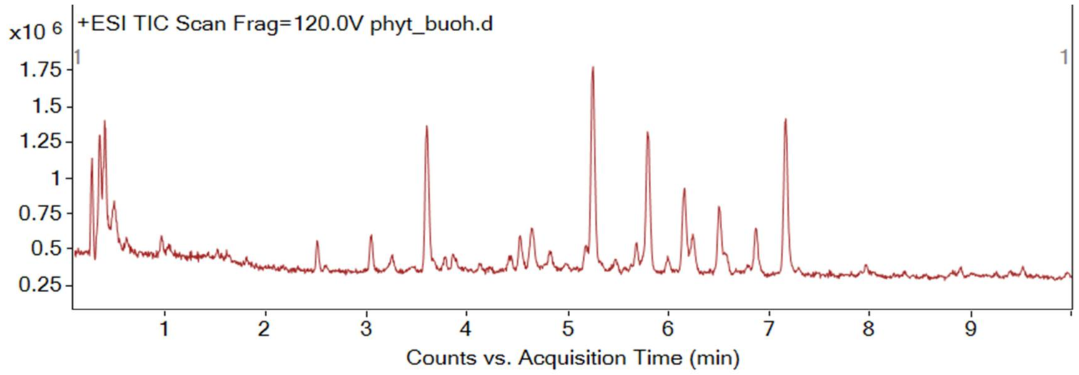

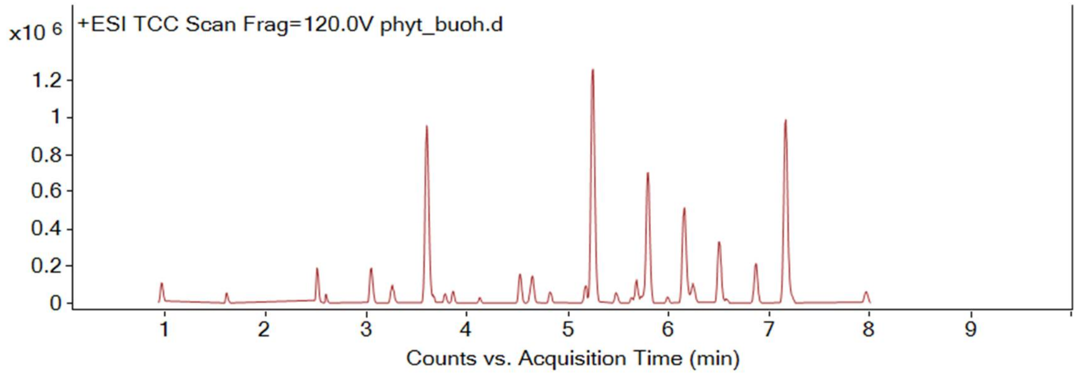

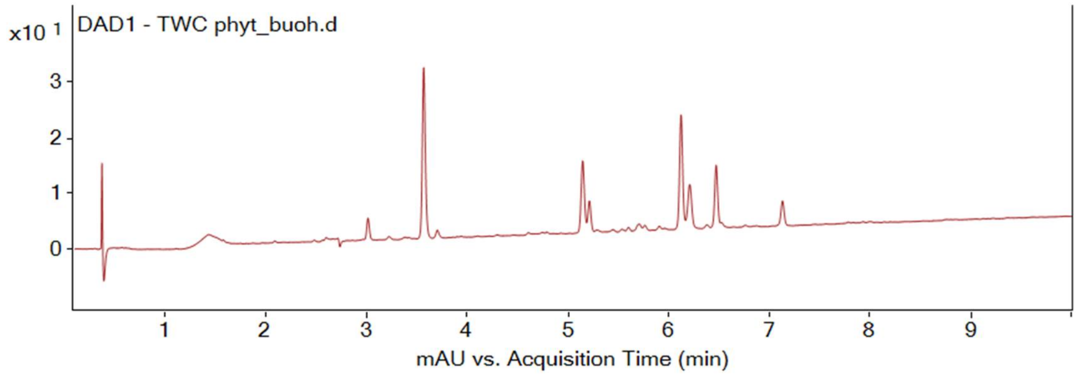

B. LC-MS ESI-

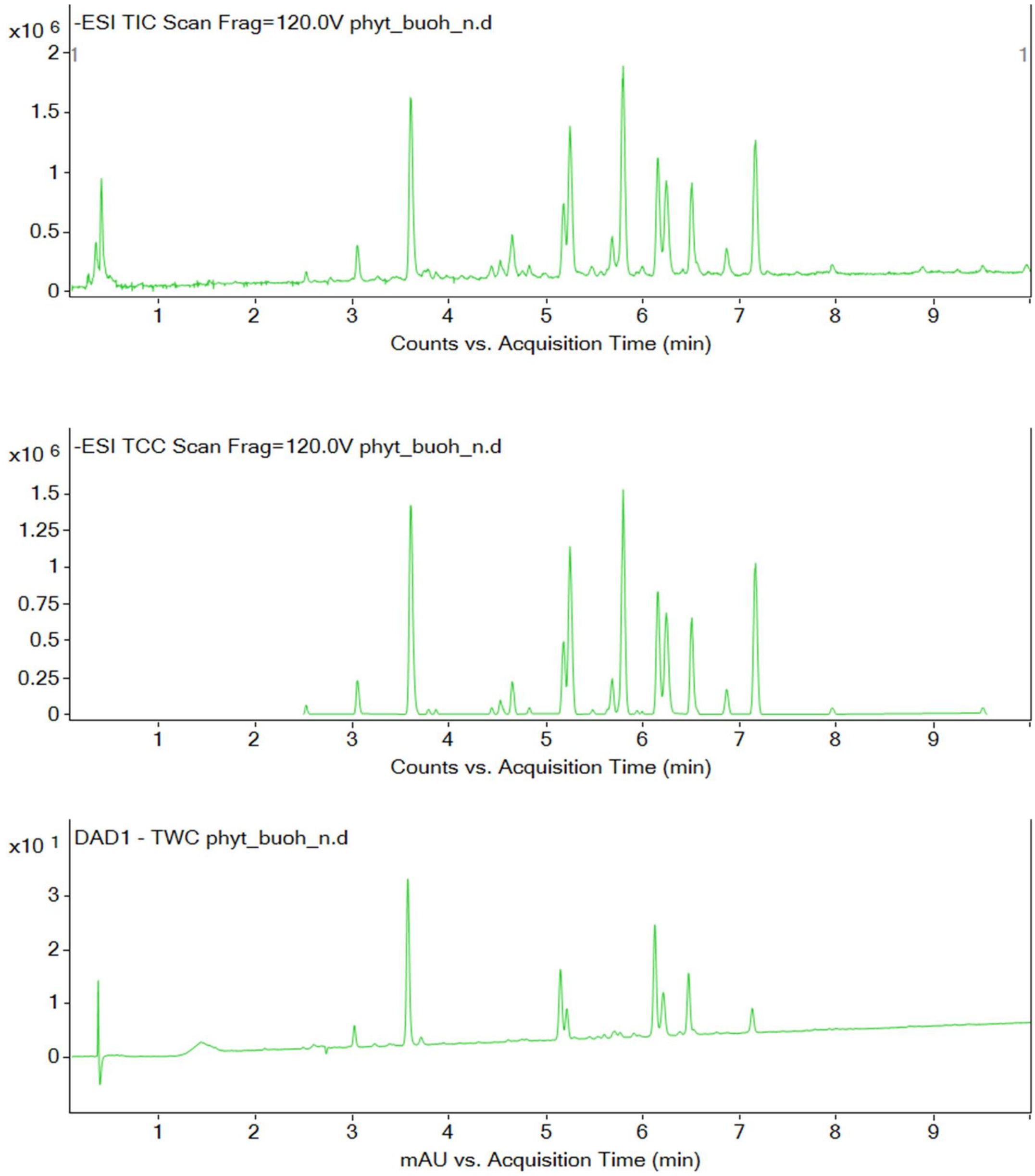
x10 6
-ESI TIC Scan Frag=120.0V phyt_buoh_n.d
Counts vs. Acquisition Time (min)
x10 6
-ESI TCC Scan Frag=120.0V phyt_buoh_n.d
Counts vs. Acquisition Time (min)
x10 1
DAD1 - TWC phyt_buoh_n.d
mAU vs. Acquisition Time (min)

S1.6 LC-ESI-MS of BuOH extract of *Crepis rubra* in positive (A) and negative ion (B) modes.

A. LC-MS ESI+

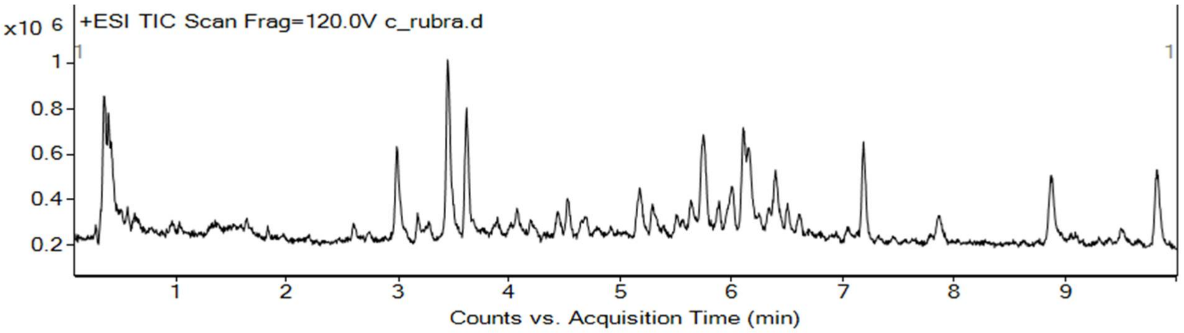

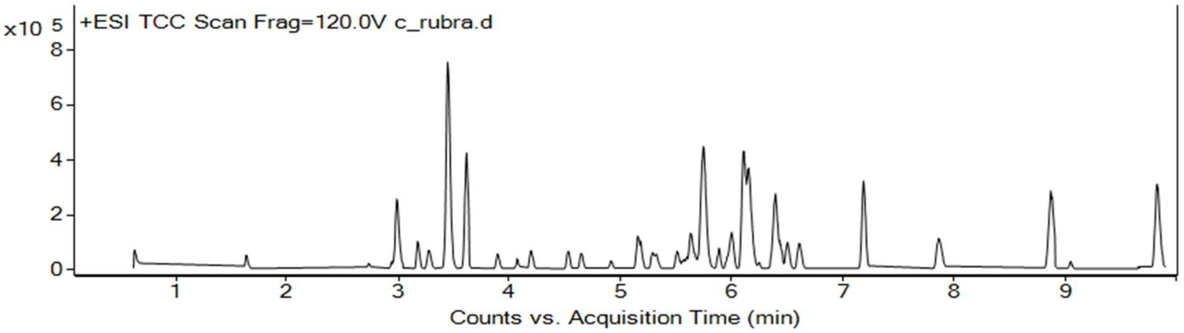

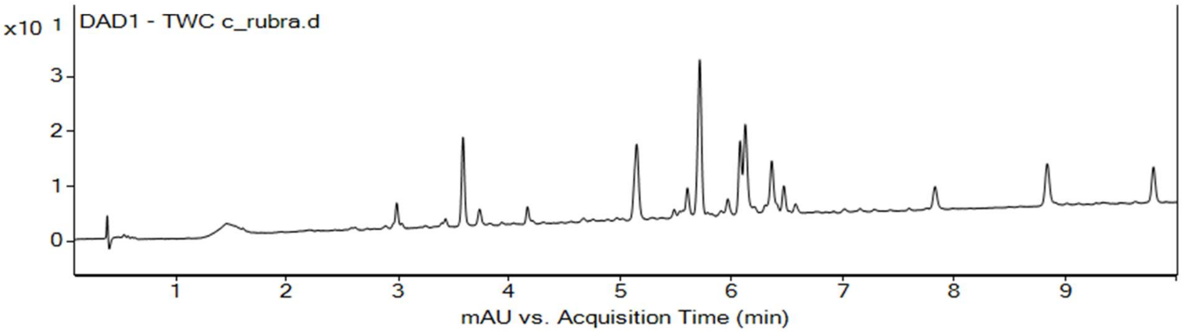

B. LC-MS ESI-

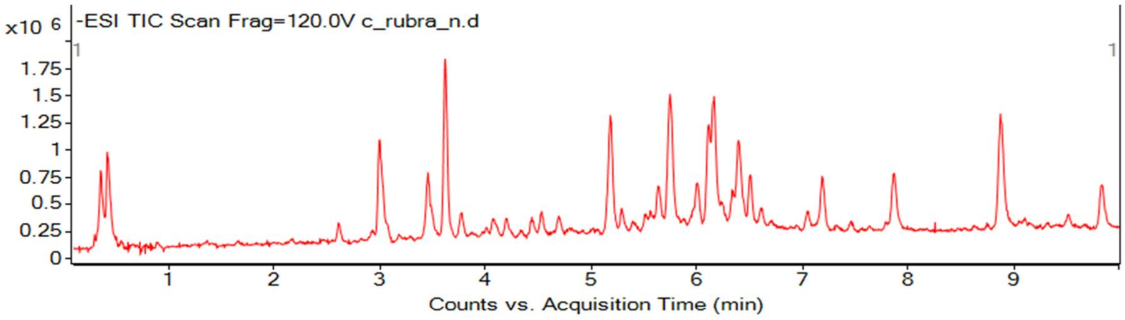
x10 6
-ESI TIC Scan Frag=120.0V c_rubra_n.d
1.75
1.5
1.25
1
0.75
0.5
0.25
0
1 2 3 4 5 6 7 8 9
Counts vs. Acquisition Time (min)

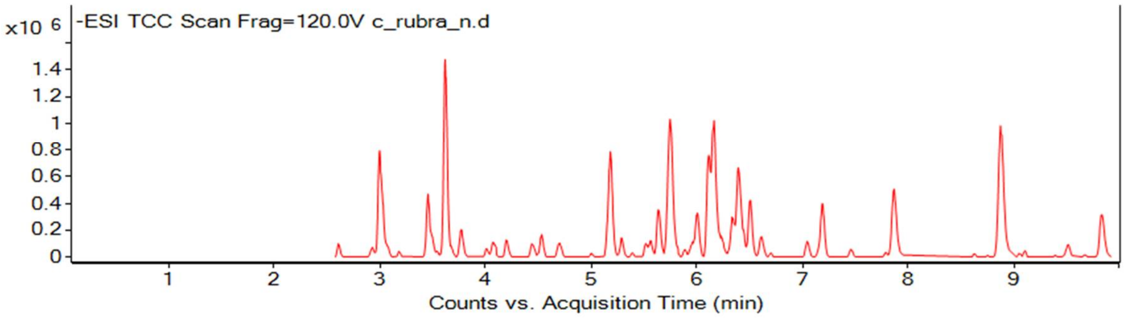
x10 6
-ESI TCC Scan Frag=120.0V c_rubra_n.d
1.4
1.2
1
0.8
0.6
0.4
0.2
0
1 2 3 4 5 6 7 8 9
Counts vs. Acquisition Time (min)

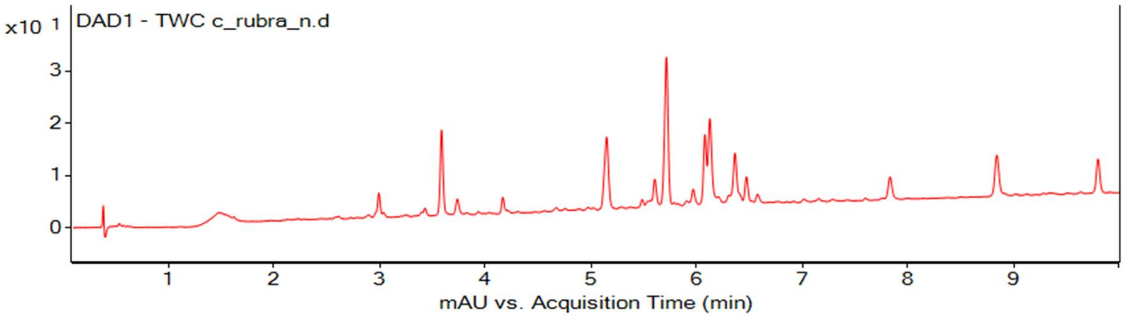
x10 1
DAD1 - TWC c_rubra_n.d
3
2
1
0
1 2 3 4 5 6 7 8 9
mAU vs. Acquisition Time (min)

S1.7 LC-ESI-MS of BuOH extract of *Crepis foetida* in positive (A) and negative ion (B) modes.

A. LC-MS ESI+

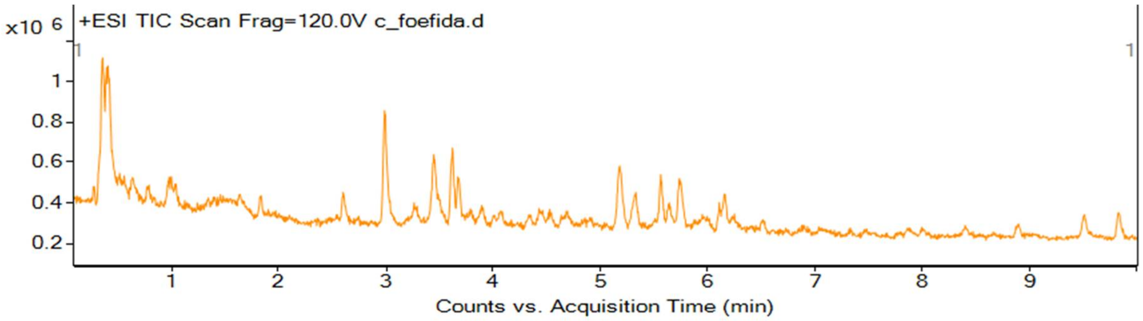

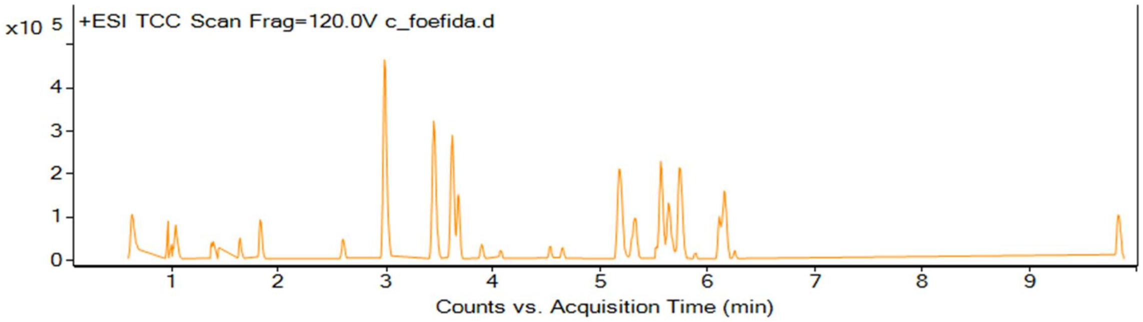

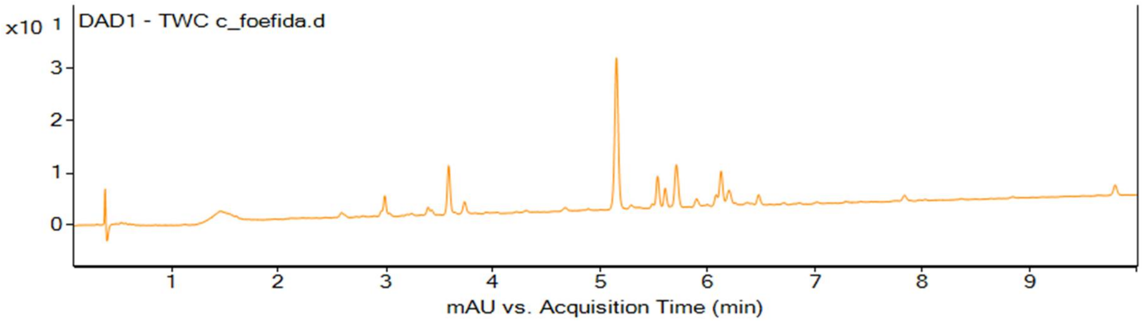

B. LC-MS ESI-

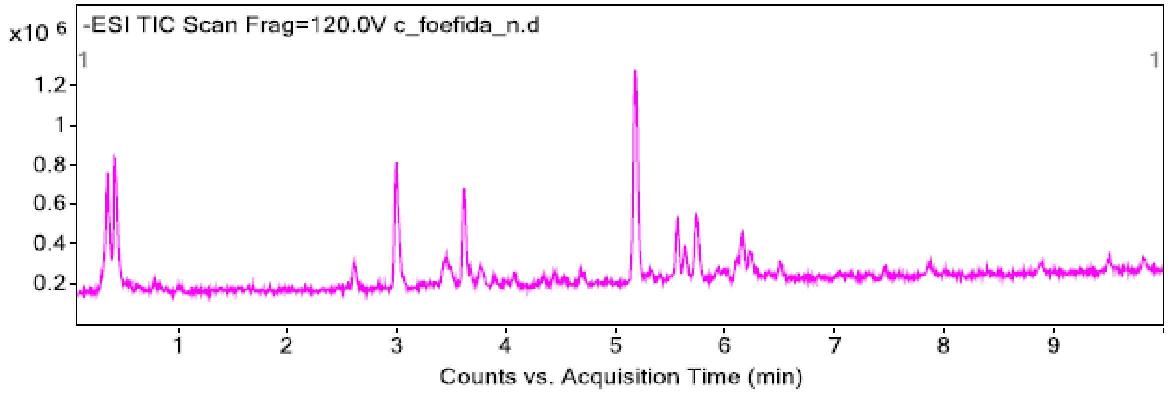
x10 6
-ESI TIC Scan Frag=120.0V c_foefida_n.d
1.2
1
0.8
0.6
0.4
0.2
1 2 3 4 5 6 7 8 9
Counts vs. Acquisition Time (min)

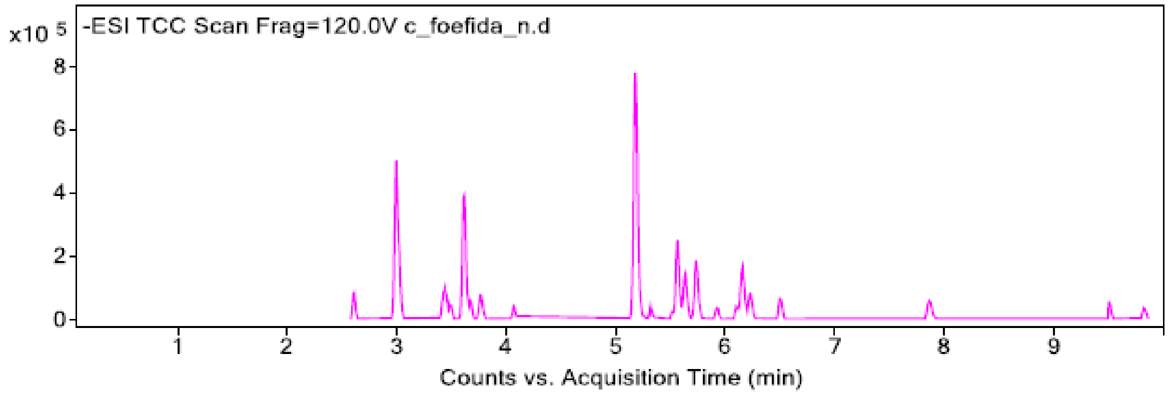
x10 5
-ESI TCC Scan Frag=120.0V c_foefida_n.d
8
6
4
2
0
1 2 3 4 5 6 7 8 9
Counts vs. Acquisition Time (min)

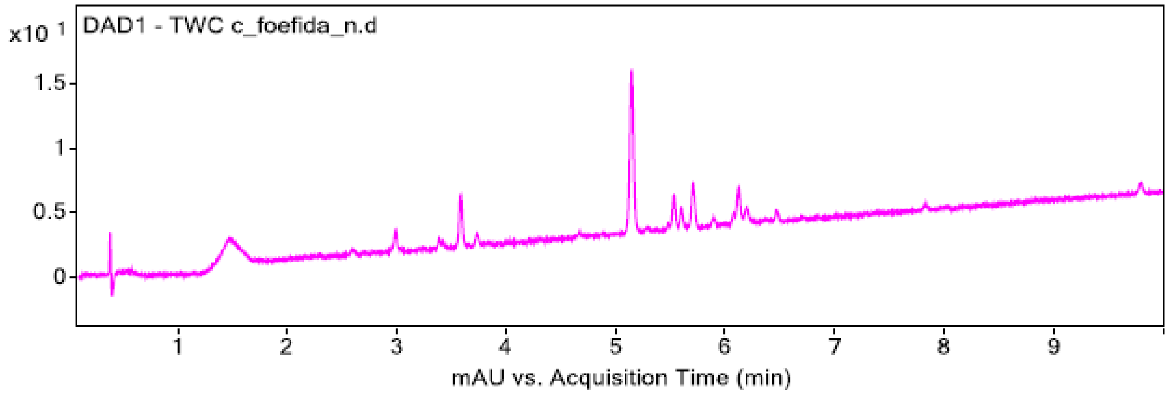
x10 1
DAD1 - TWC c_foefida_n.d
1.5
1
0.5
0
1 2 3 4 5 6 7 8 9
mAU vs. Acquisition Time (min)

S1.8 LC-ESI-MS of BuOH extract of *Crepis incana* in positive (A) and negative ion (B) modes.

A. LC-MS ESI+

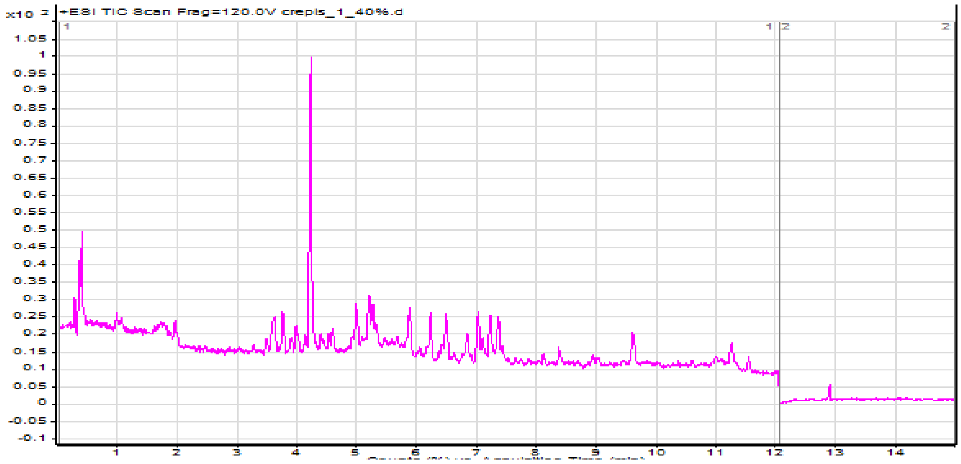

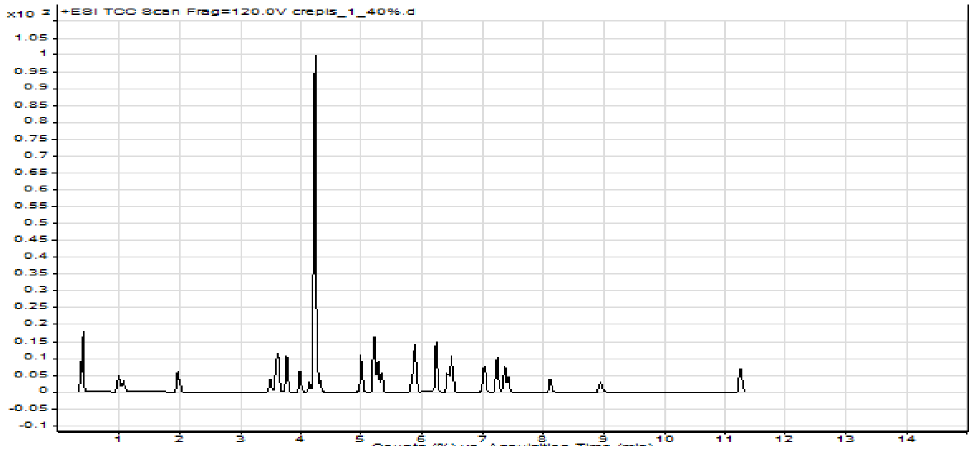

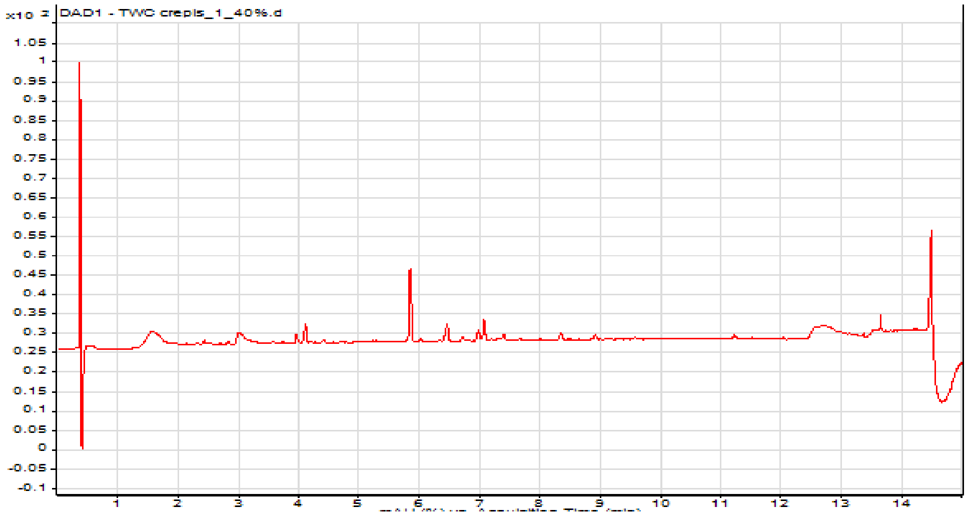

B. LC-MS ESI-

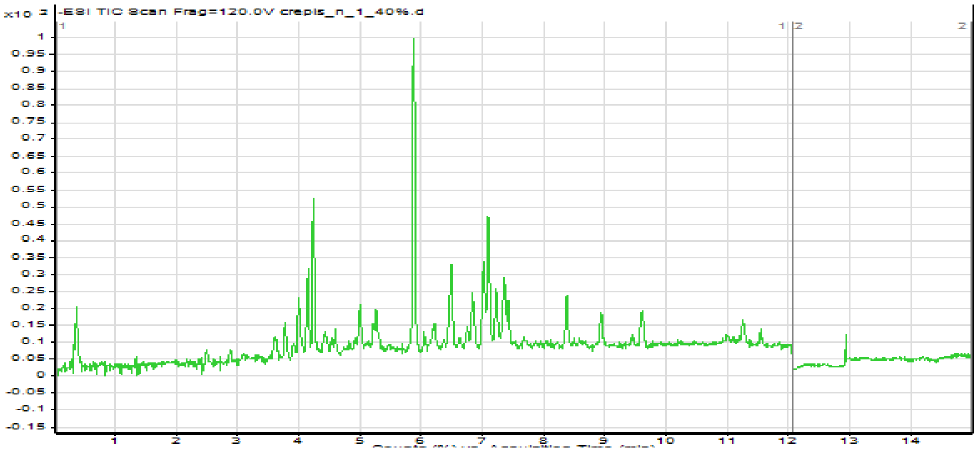
-ESI TIC Scan Frag=120.0V crepis_n_1_40%.d

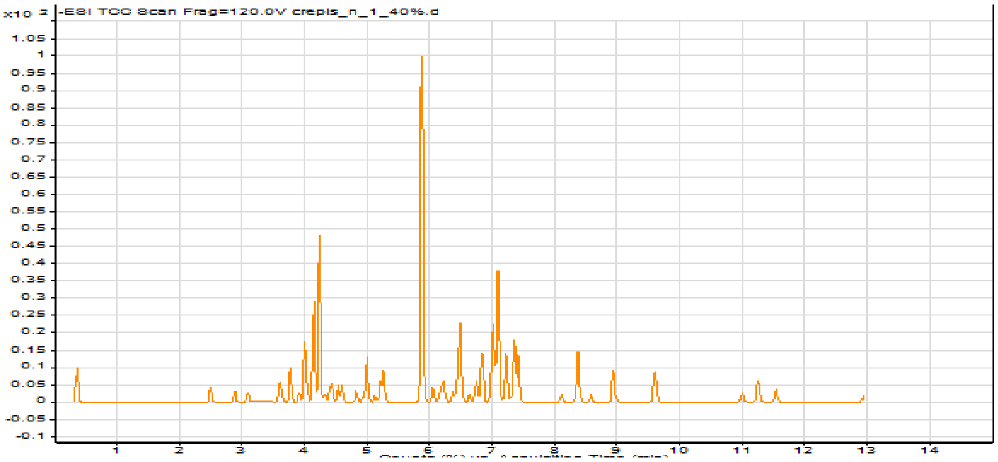
-ESI TCC Scan Frag=120.0V crepis_n_1_40%.d

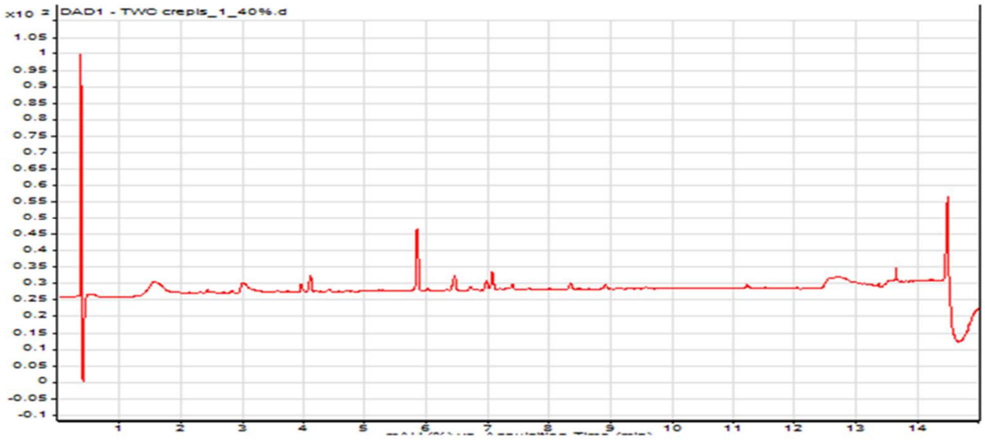
DAD1 - TWC crepis_1_40%.d

S.2 MS-MS spectrum of selected compounds in positive (A) and/or negative (B) ion mode

S.2.1 MS-MS spectrum of cichoric acid

A.

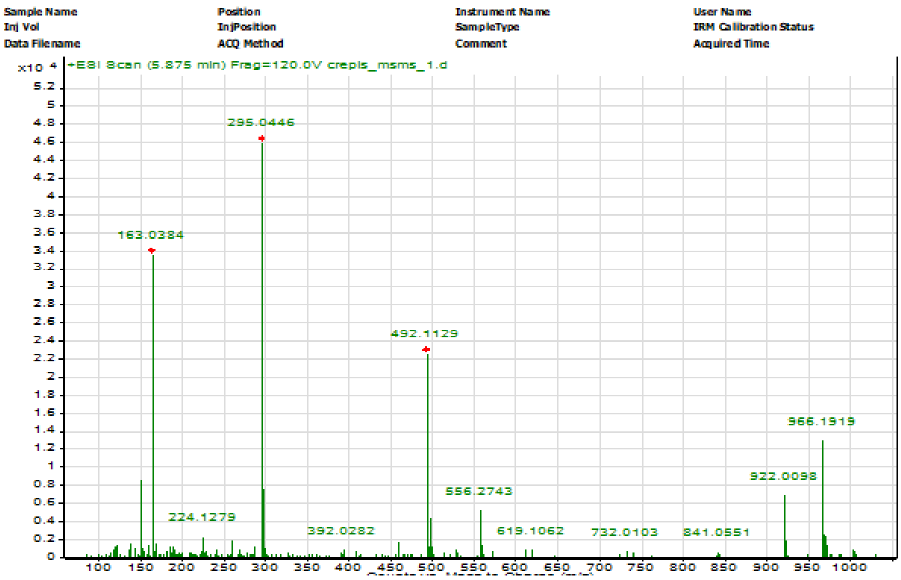

B.

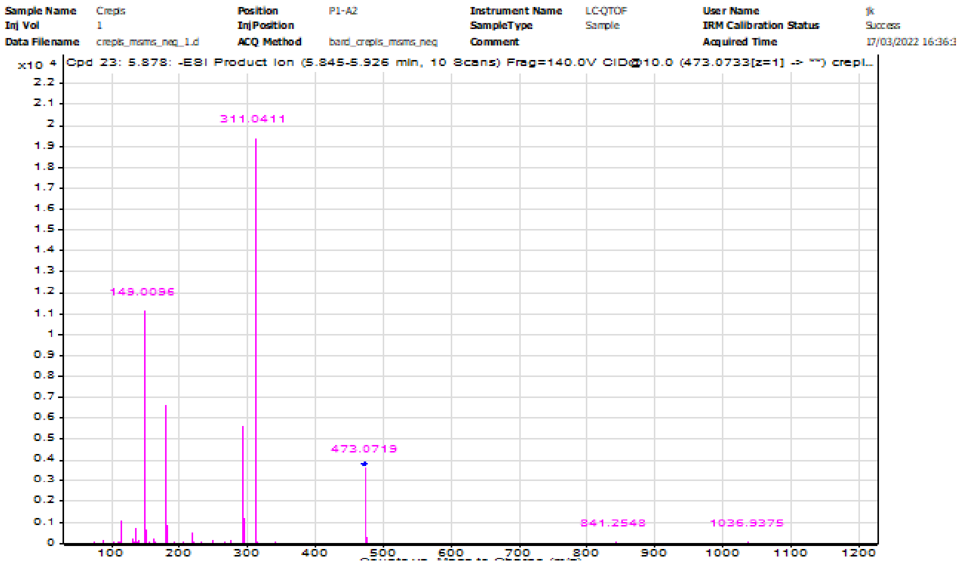

S.2.2 MS-MS spectrum of chlorogenic acid isomer

A.

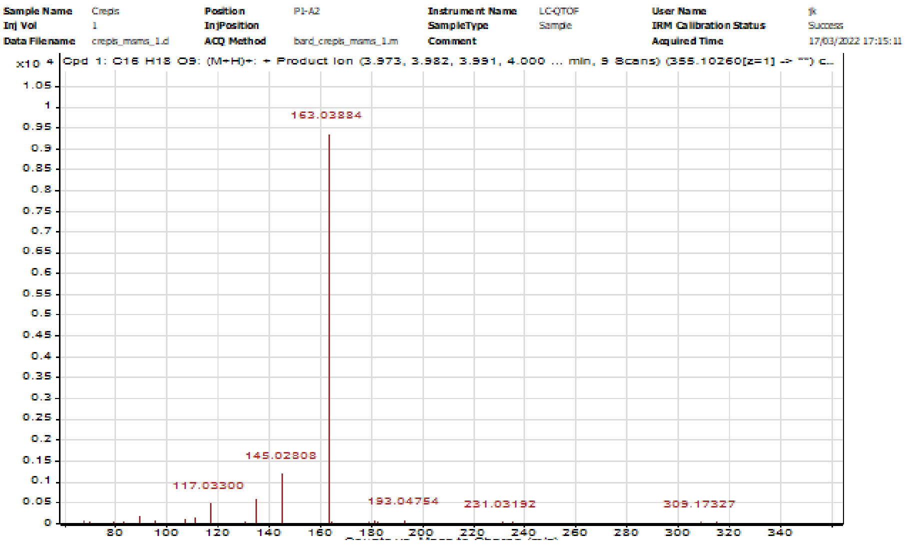

B.

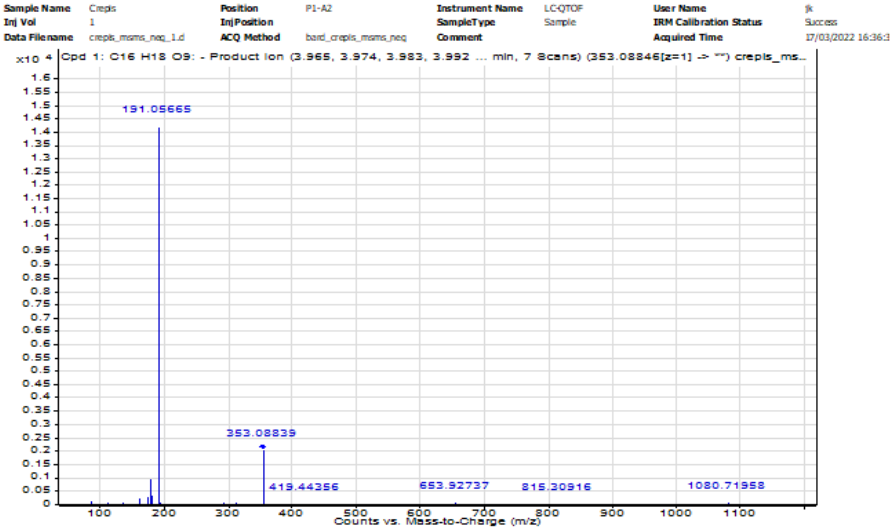

S.2.3 MS-MS spectrum of luteolin 7-glucoside (Cynaroside)

A.

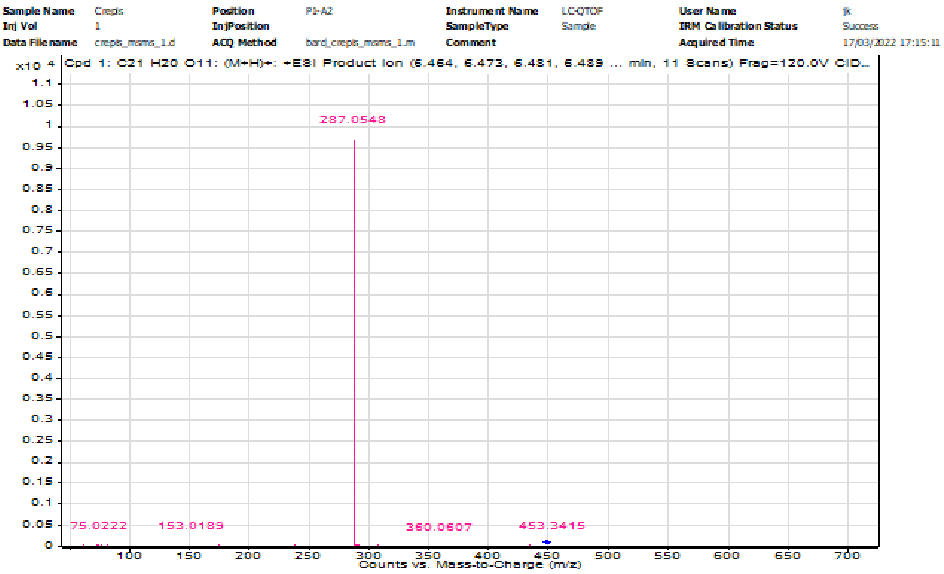

B.

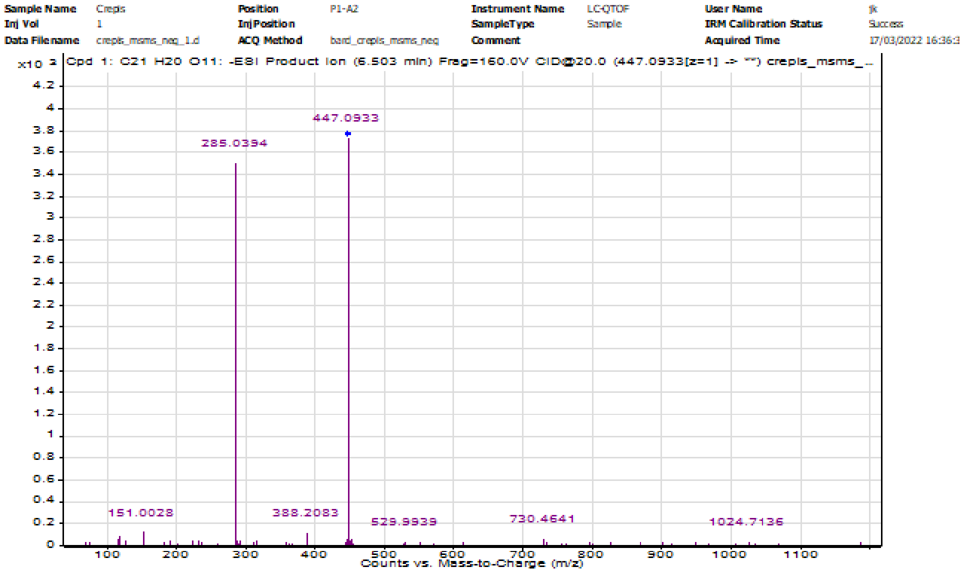

S.2.4 MS-MS spectrum of Di-caffeoylquinic acid isomer

B.

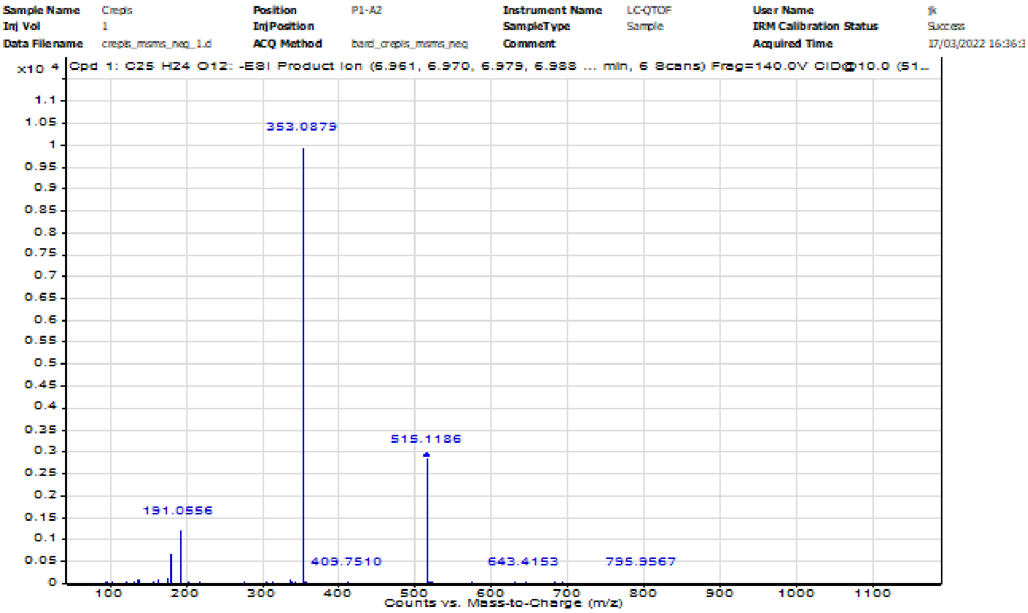

S.2.5 MS-MS spectrum of luteolin

A.

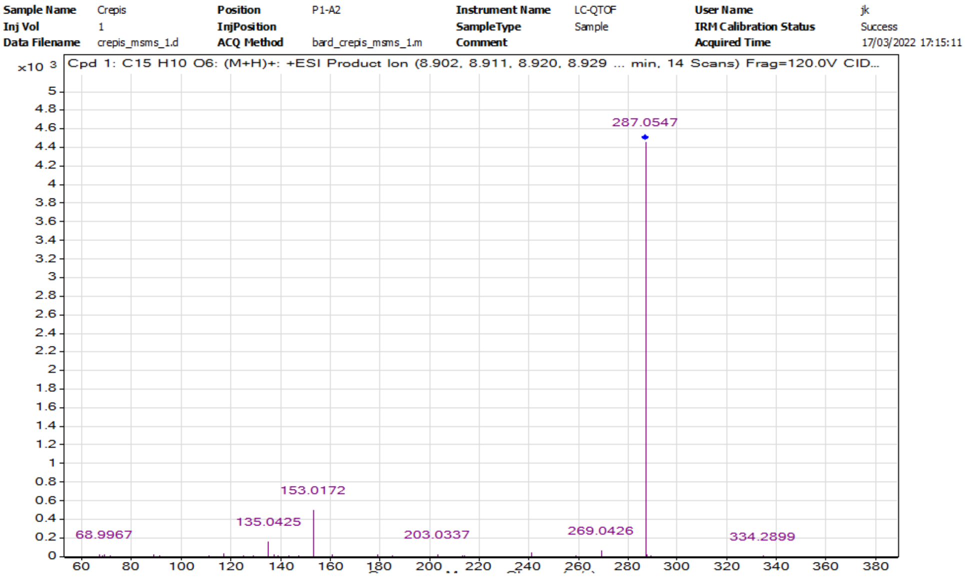

B.

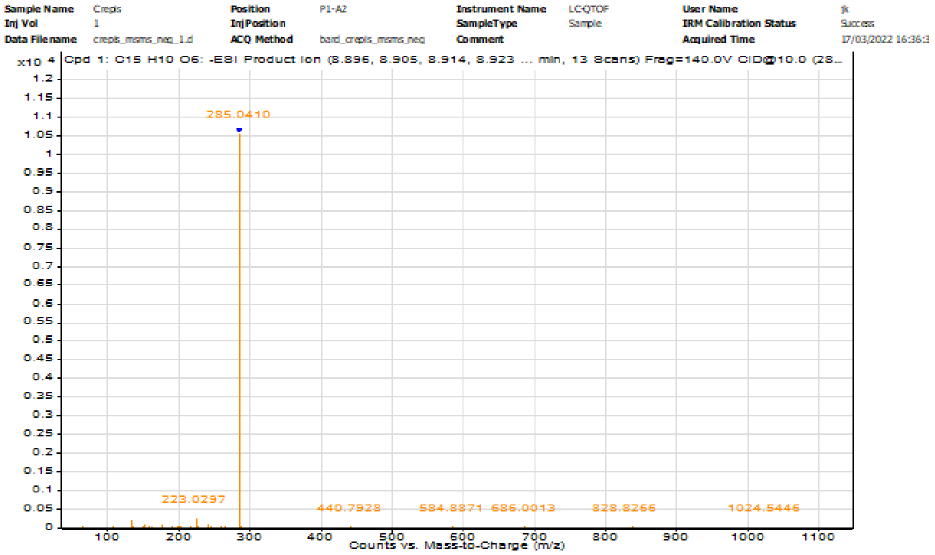
Sample Name Crepis
Inj Vol 1
Data Filename crepis_msms_neg_1.d
Position P1-A2
InjPosition
ACQ Method bard_crepis_msms_neg
Instrument Name LC-QTOF
SampleType Sample
Comment
User Name jk
IRM Calibration Status Success
Acquired Time 17/03/2022 16:36:3
x10 4
Cpd 1: C15 H10 O6: -ESI Product Ion (8.896, 8.905, 8.914, 8.923 ... min, 13 Scans) Frag=140.0V CID@10.0 (28...
285.0410
223.0297
440.7928
584.8871
686.0013
828.8266
1024.5446
Counts vs. Mass-to-Charge (m/z)

S3. NMR data

S3.1 ^{1}H NMR (600 MHz, CD_3OD) spectrum of *Crepis rubra*

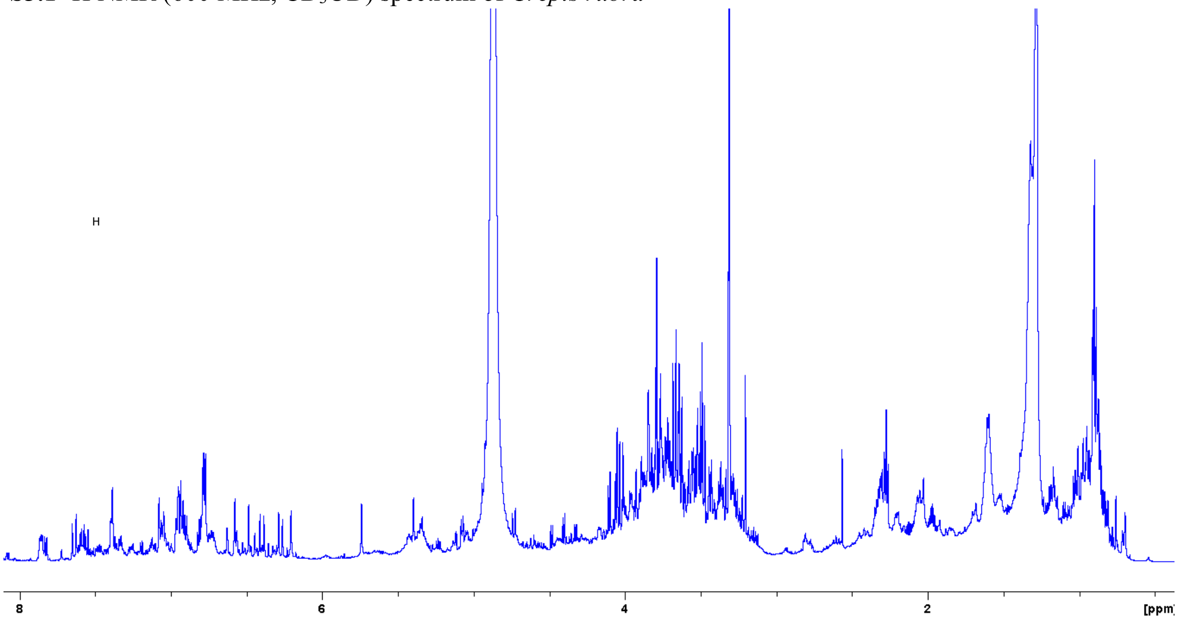

S3.2 ^{13}C NMR (150 MHz, CD_3OD) spectrum of *C. rubra*

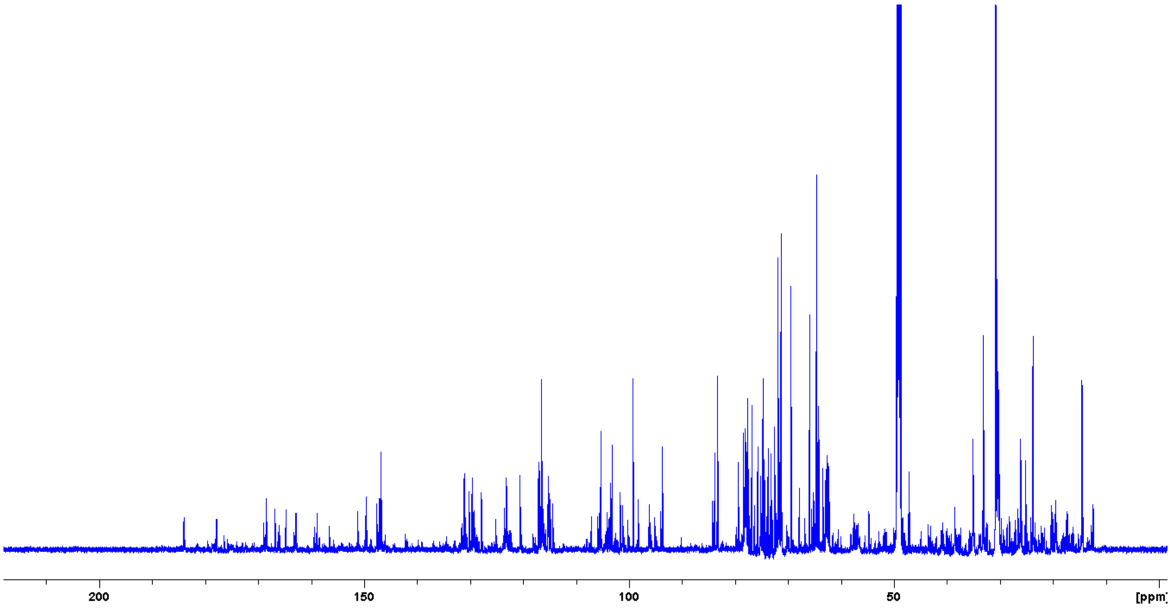

S3.3 HSQC (CD_3OD) spectrum of *C. rubra*

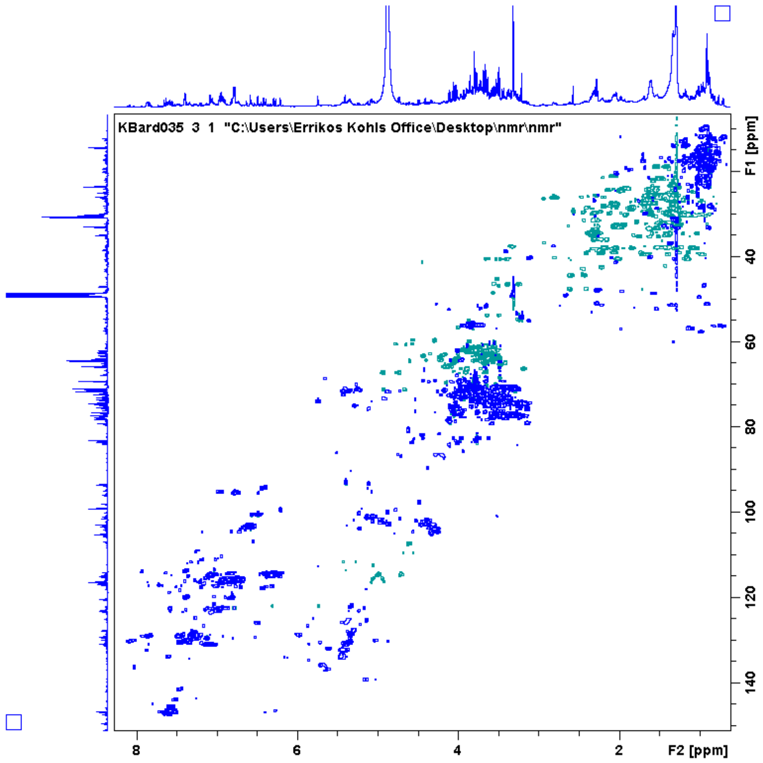

S3.4 HMBC (CD_3OD) spectrum of *C. rubra*

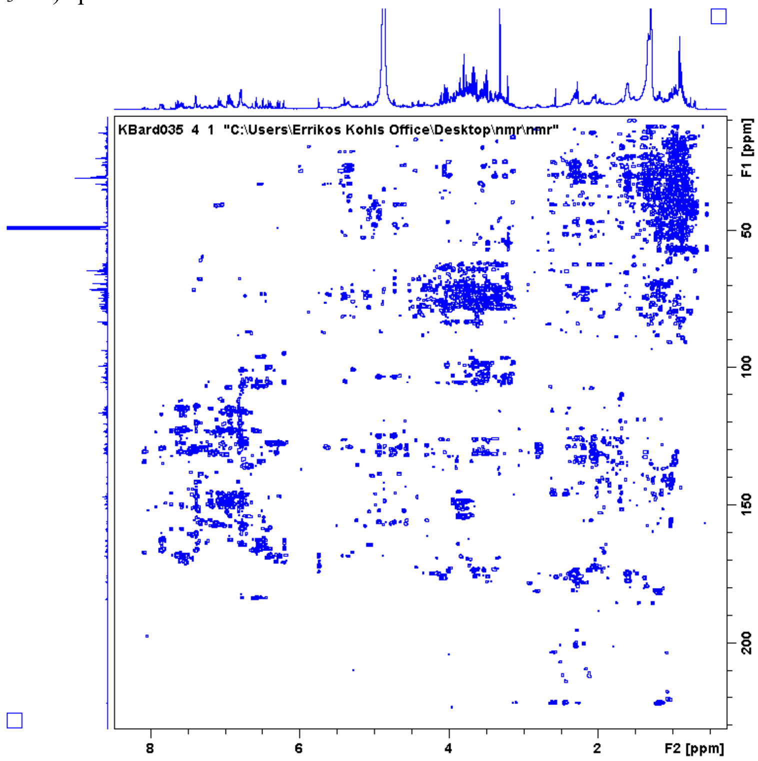

S3.5 ^{1}H NMR (600 MHz, CD_3OD) spectrum of *Crepis dioscoridis*

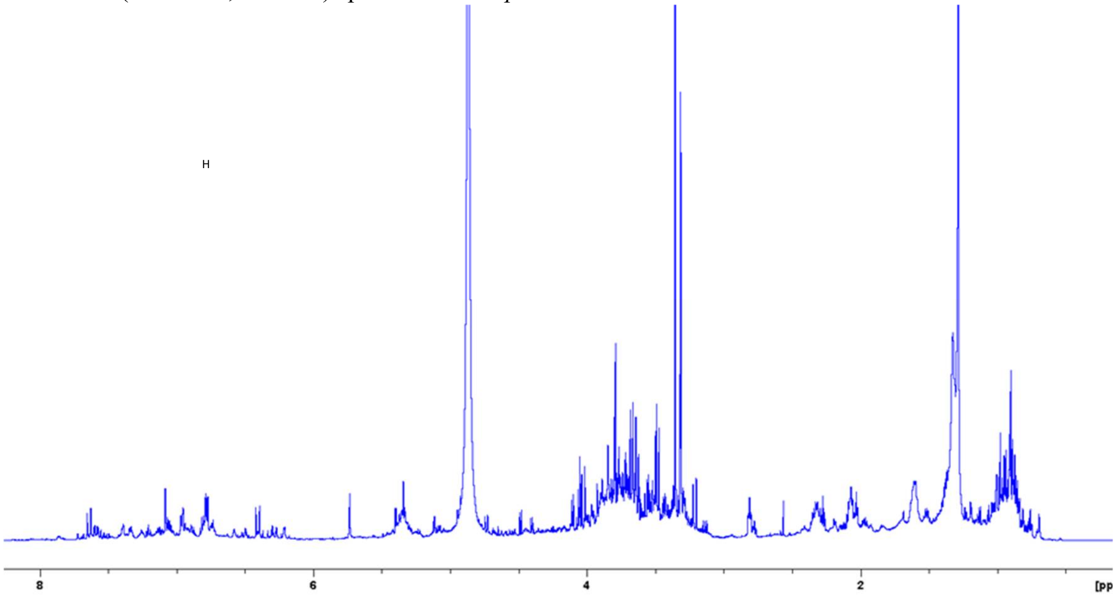

S3.6 ^{13}C NMR (150 MHz, CD_3OD) spectrum of *C. dioscoridis*

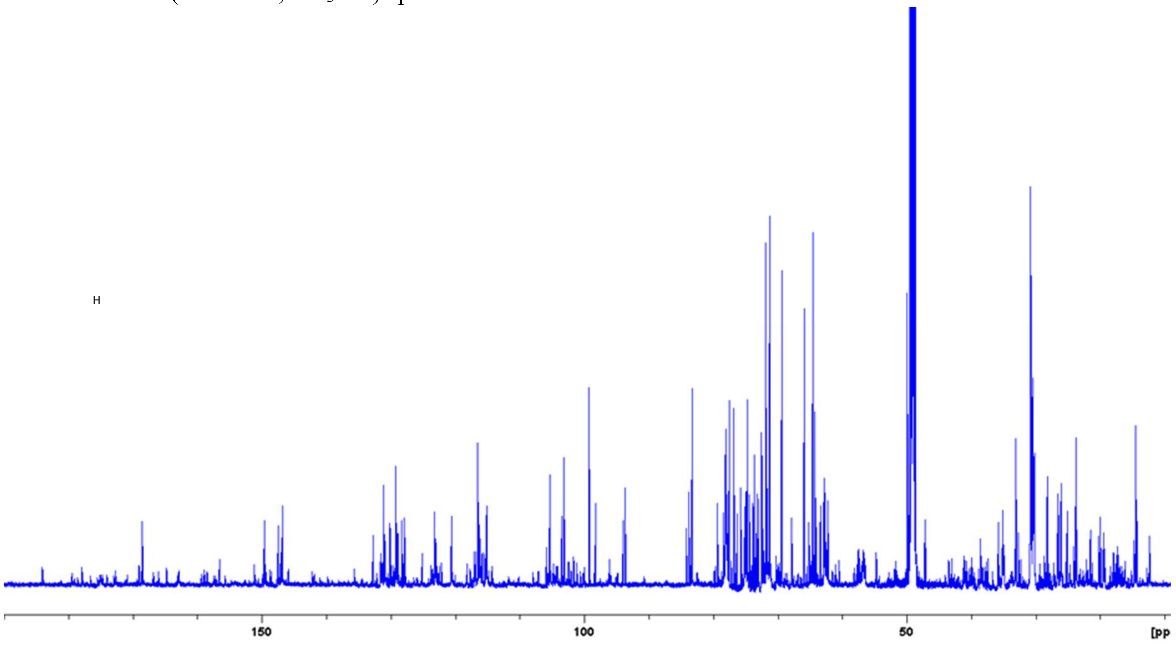

S3.7 HSQC (CD_3OD) spectrum of *C. dioscoridis*

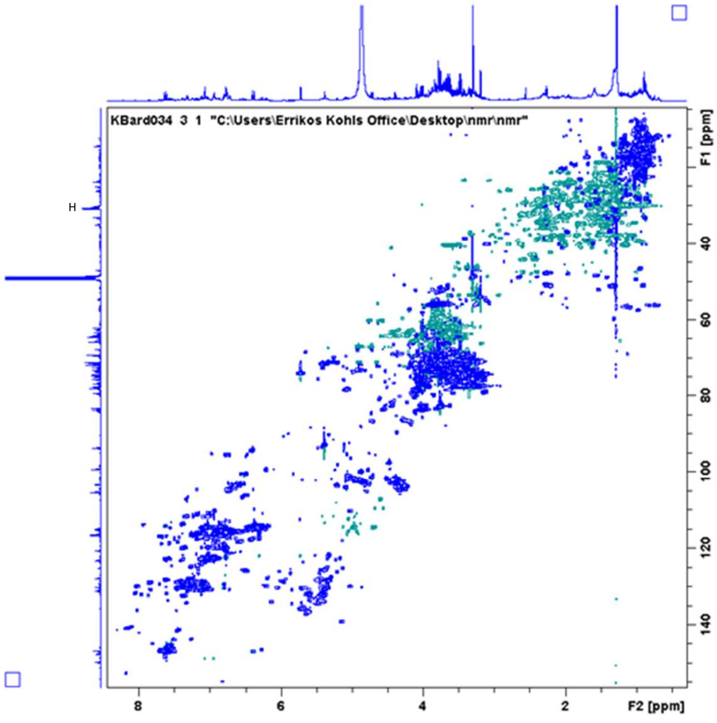

S3.8 HMBC (CD_3OD) spectrum of *C. dioscoridis*

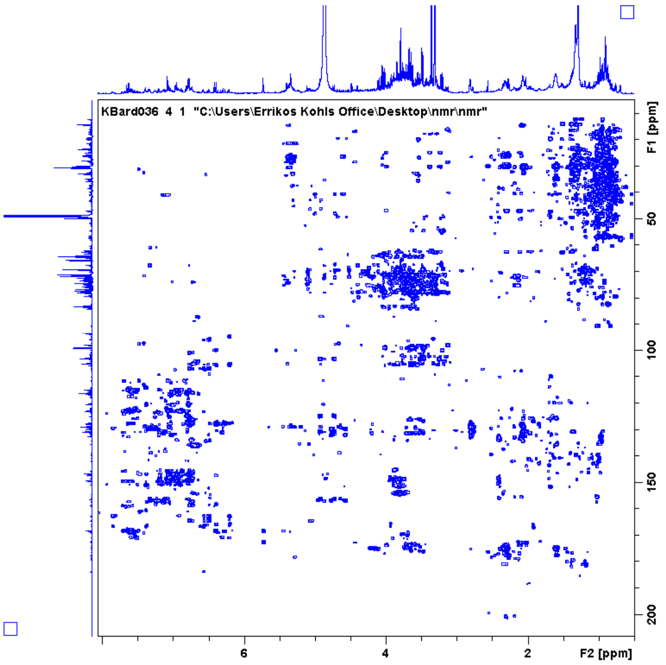

S3.9 ^{1}H NMR (600 MHz, CD_3OD) spectrum of *Crepis foetida*

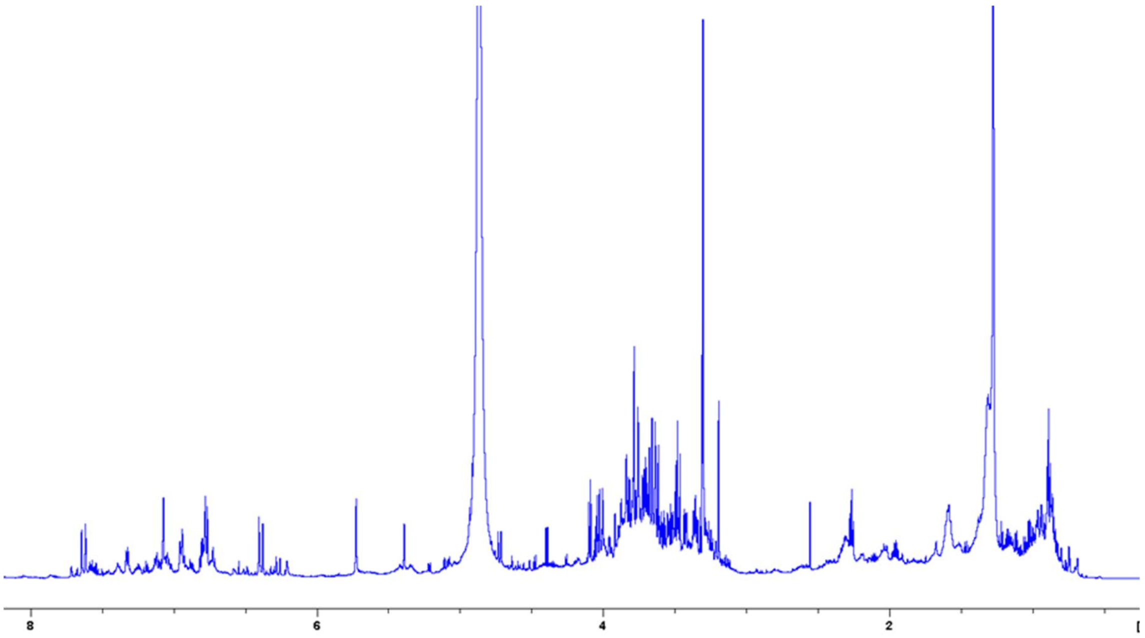

S3.10 ^{13}C (150 MHz, CD_3OD) spectrum of *C. foetida*

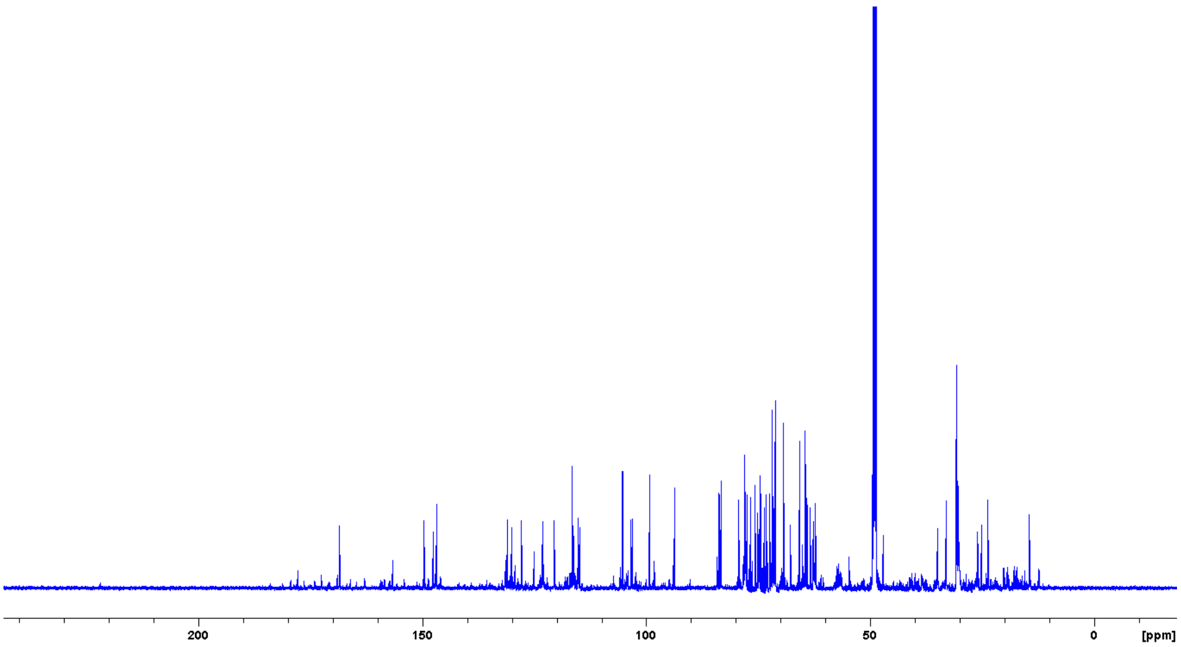

S3.11 HSQC (CD_3OD) spectrum of *C. foetida*

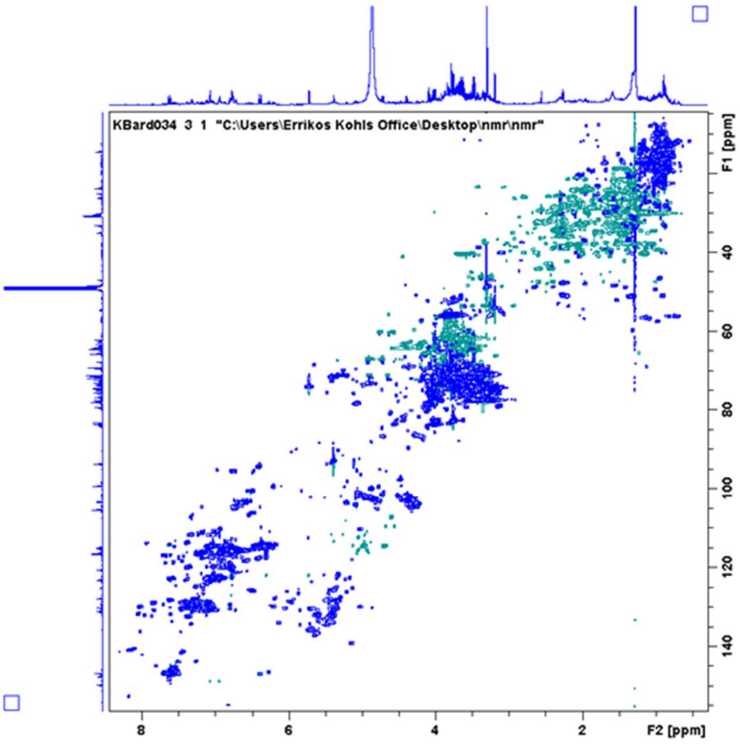

S3.12. HMBC (CD_3OD) spectrum of *C. foetida*

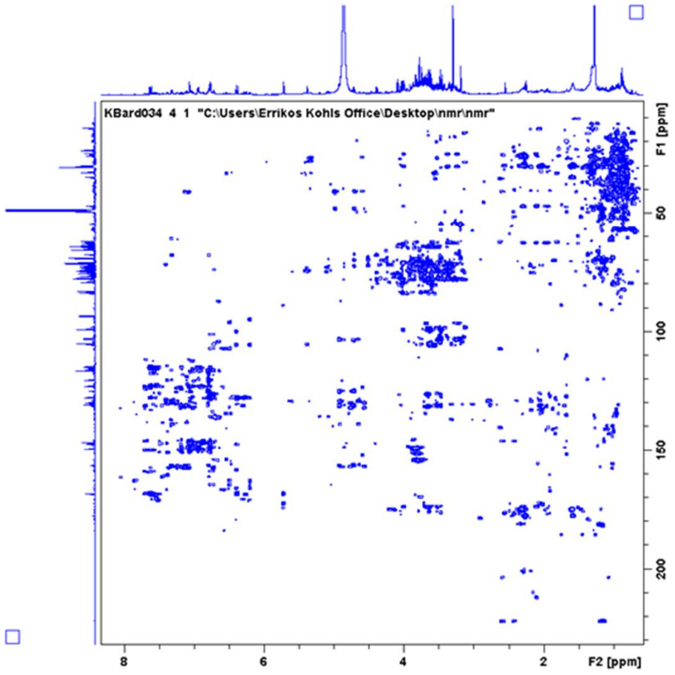

S3.13 ^{1}H NMR (600 MHz, CD_3OD) spectrum of *Crepis commutata*

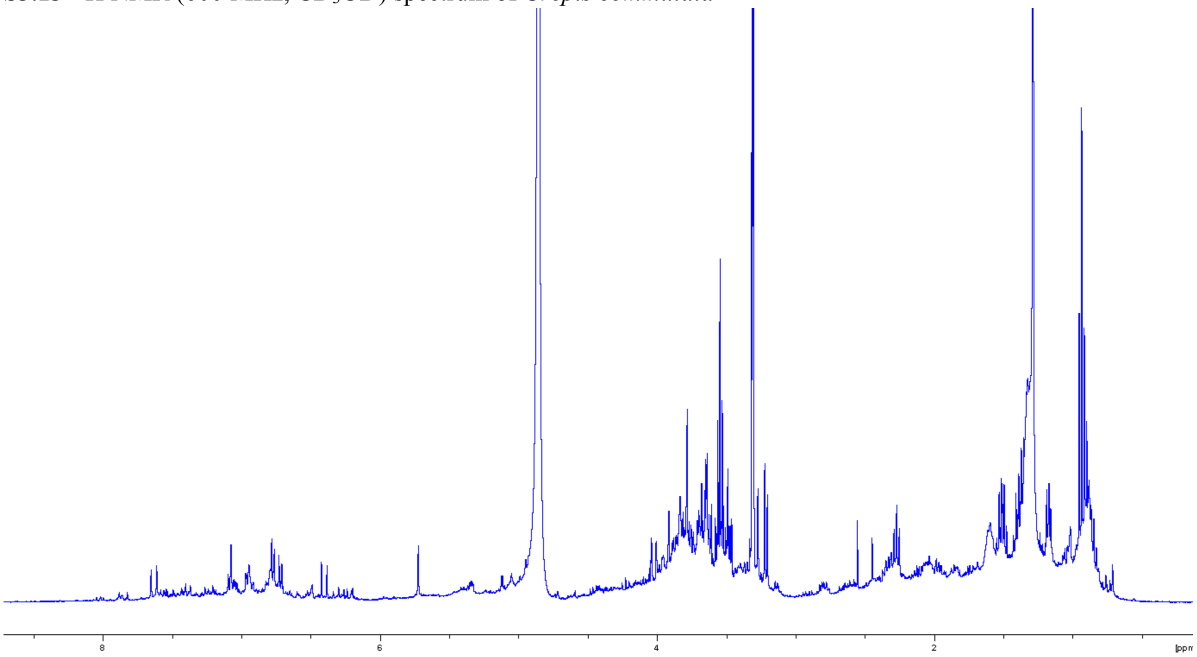

S3.14 ^{13}C NMR (150 MHz, CD_3OD) spectrum of *C. commutata*

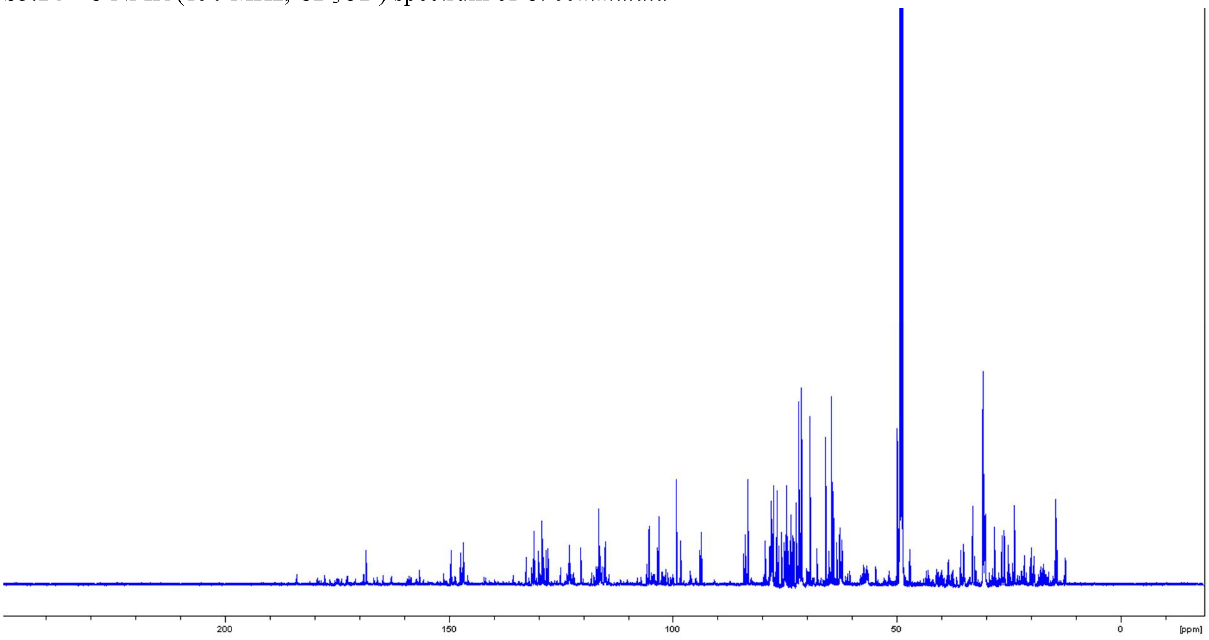

S3.15 HSQC (CD_3OD) spectrum of *C. commutata*

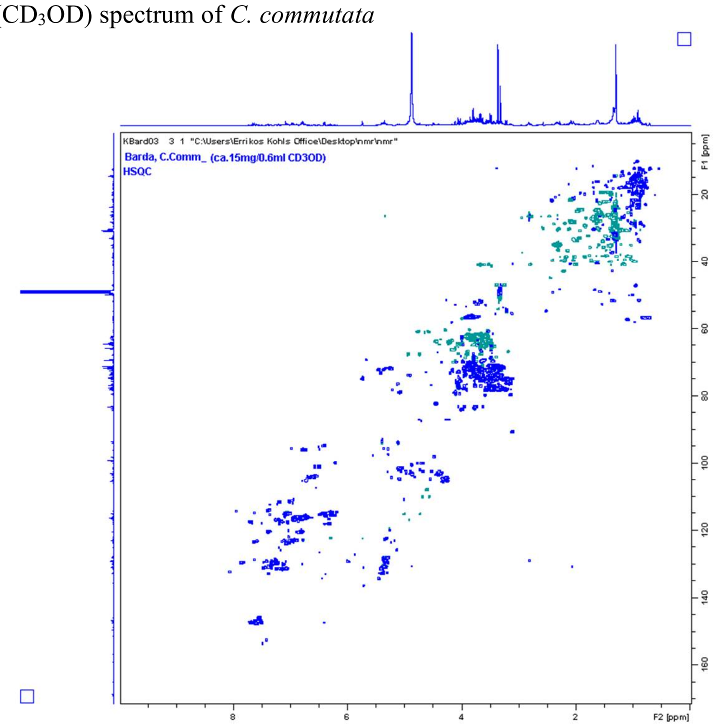

S3.16 HMBC (CD_3OD) spectrum of *C. commutata*

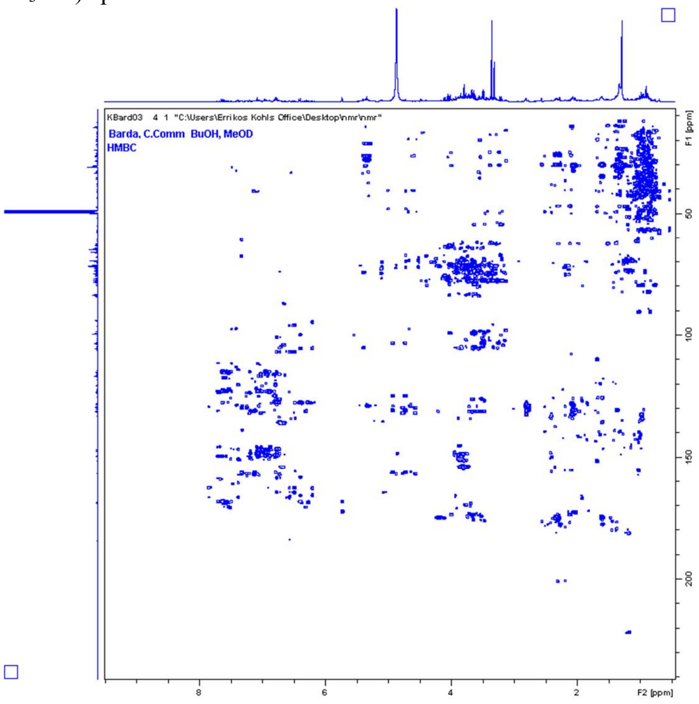

S3.17 ^{1}H NMR (600 MHz, CD_3OD) spectrum of *Phitosia crocifolia*

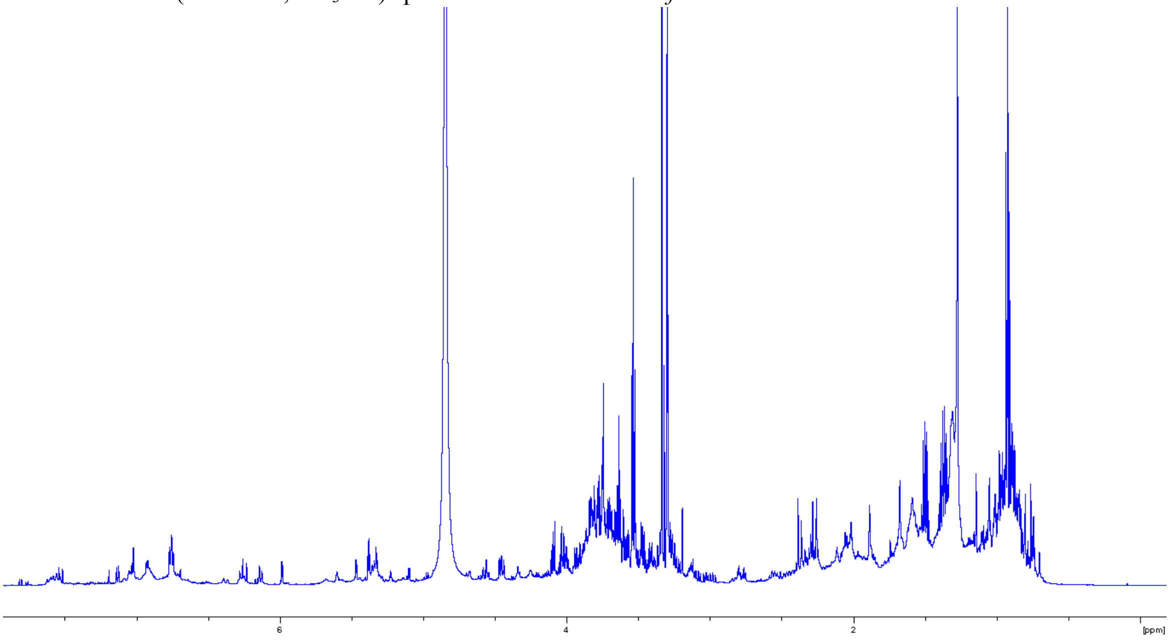

S3.18 ^{13}C NMR (150 MHz, CD_3OD) spectrum of *P. crocifolia*

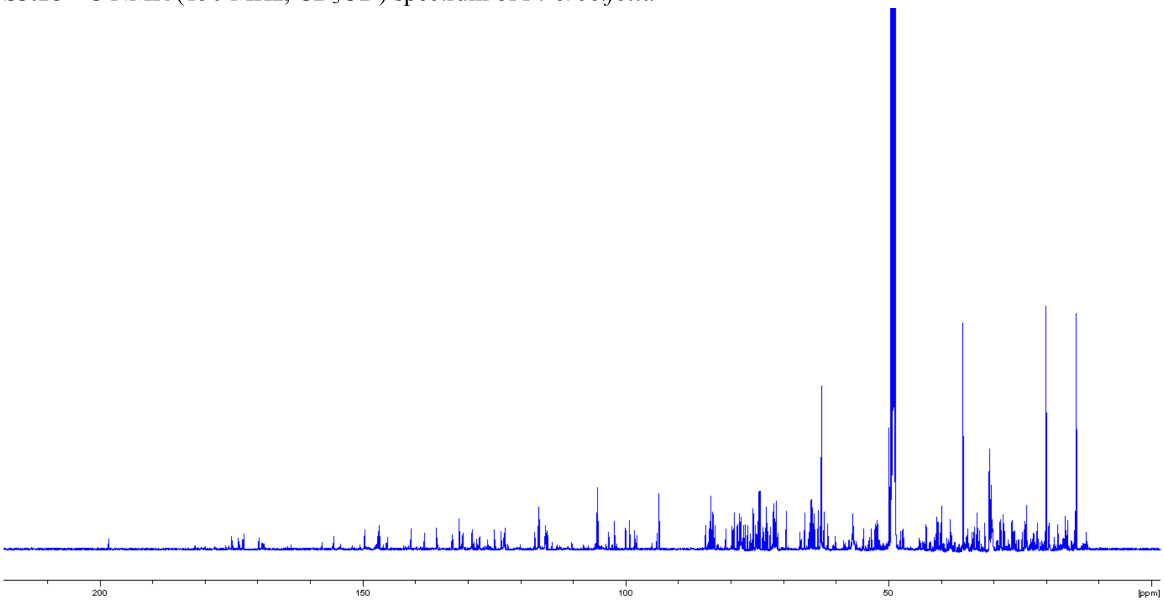

S3.19 HSQC (CD_3OD) spectrum of *P. crocifolia*

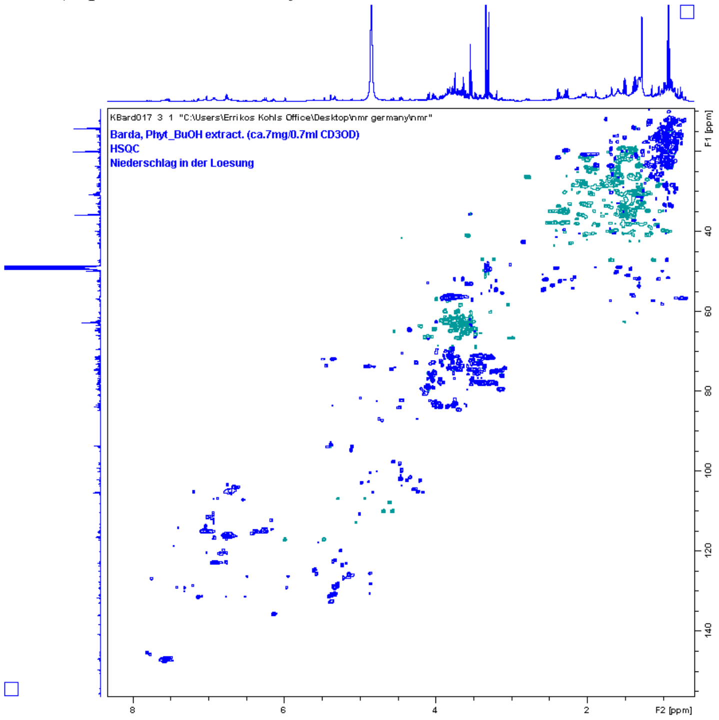

S3.20 HMBC (CD_3OD) spectrum of *P. crocifolia*

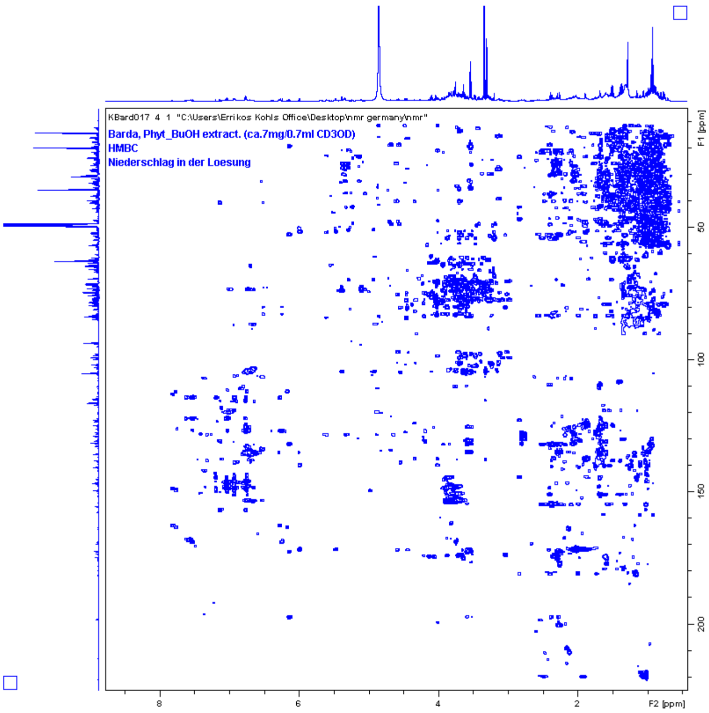

S3.21 ^{1}H NMR (600 MHz, CD_3OD) spectrum of *Crepis incana*

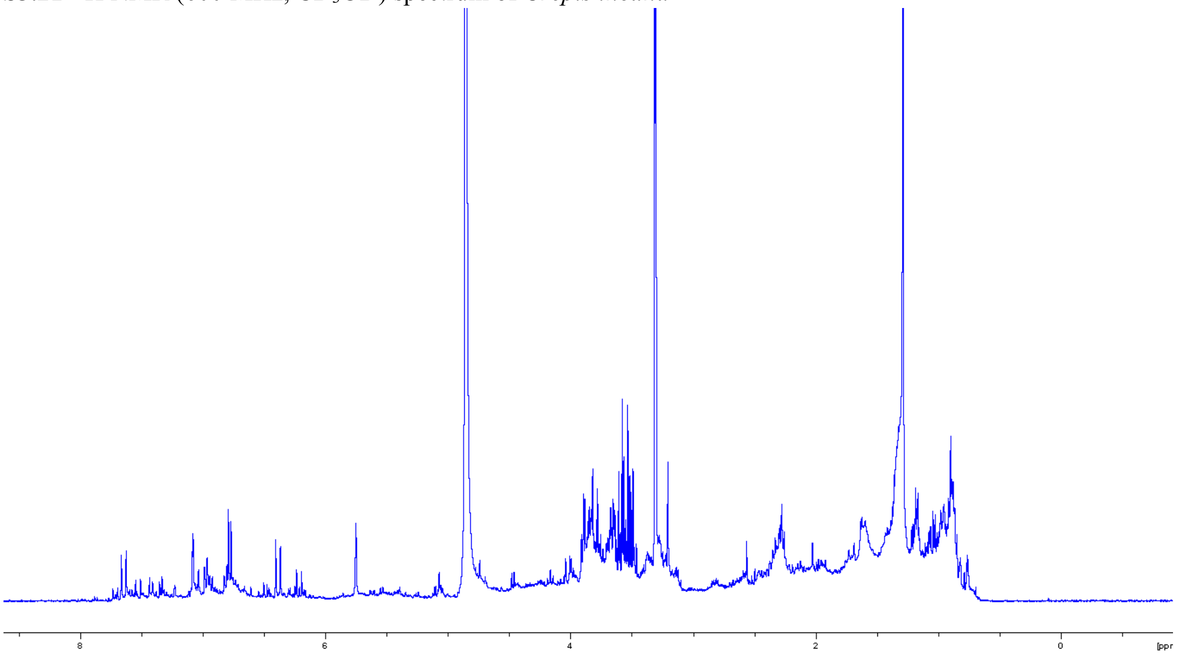

S3.22 ^{13}C NMR (150 MHz, CD_3OD) spectrum of *C. incana*

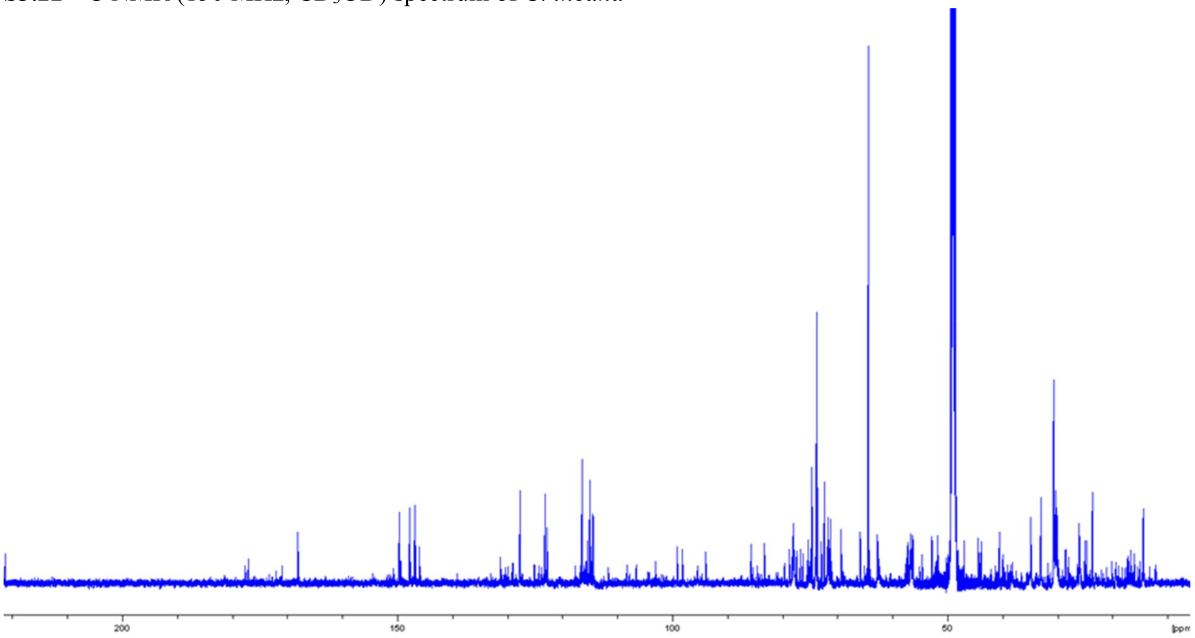

S3.23 HSQC (CD_3OD) spectrum of *C. incana*

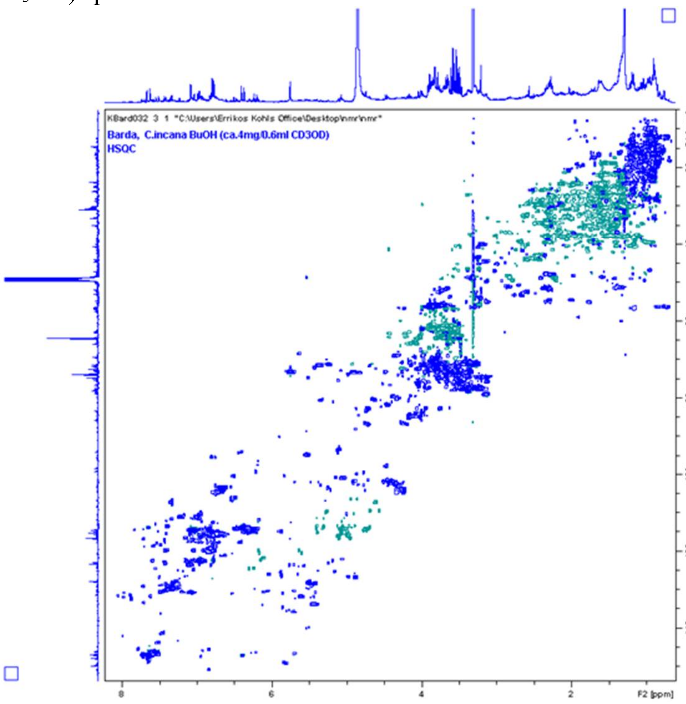

S3.24 HMBC (CD_3OD) spectrum of *C. incana*

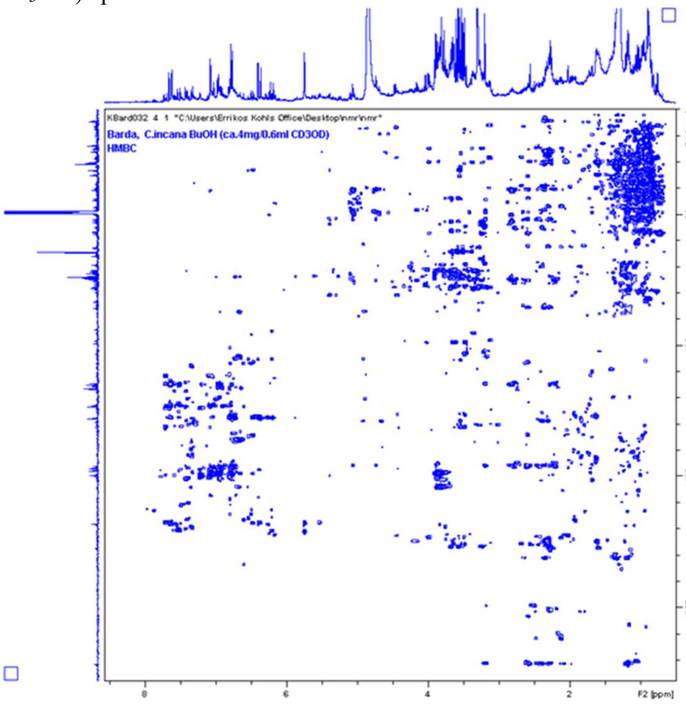

S3.25 ^{1}H NMR (600 MHz, CD_3OD) spectrum of *Crepis heldreichiana*

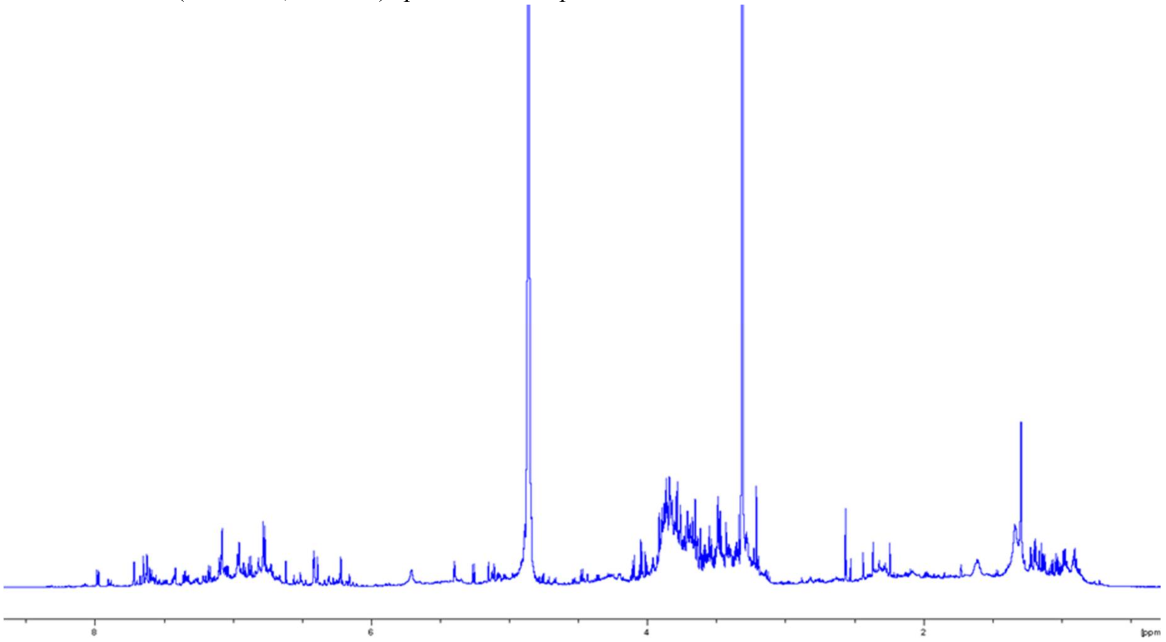

S3.26 ^{13}C NMR (150 MHz, CD_3OD) spectrum of *C. heldreichiana*

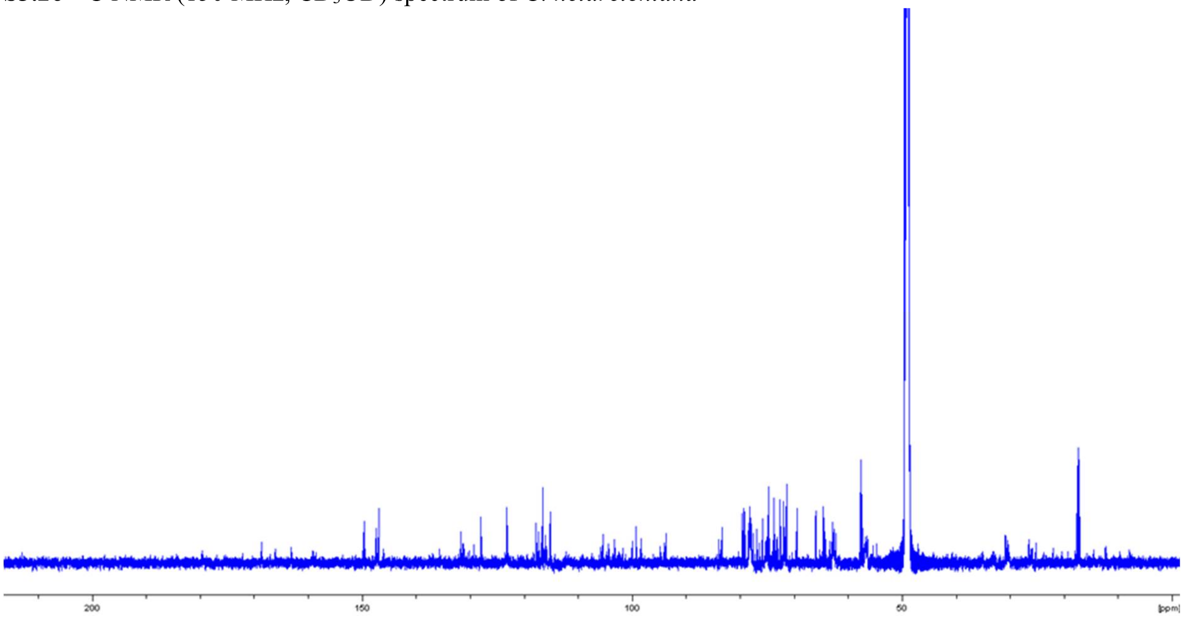

S3.27 HSQC (CD_3OD) spectrum of *C. heldreichiana*

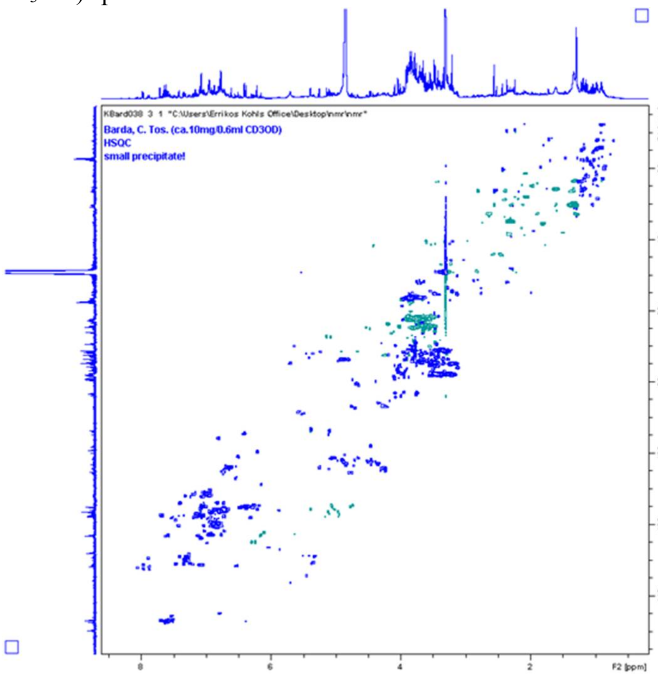

S3.28 HMBC (CD_3OD) spectrum of *C. heldreichiana*

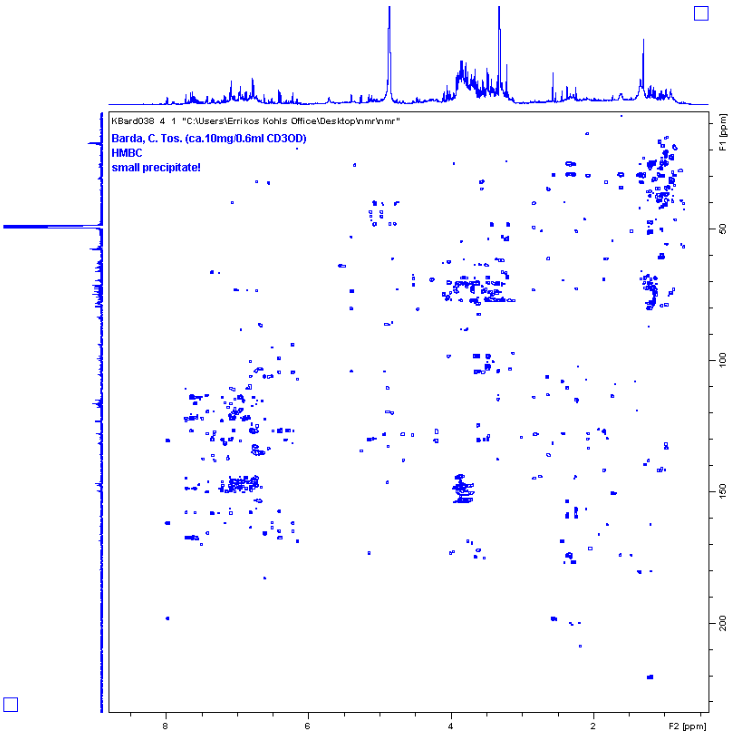

Supplement: Supplementary file 1 [file molecules-27-06173-s001.zip › molecules-1864457-supplementary.pdf]
